# Spatiotemporal Multi-Omics and Compartment-Resolved Metabolomic Analyses Reveal the Mechanisms and Core Regulatory Networks of Cuticular Wax Formation in Blueberry Fruits

**DOI:** 10.3390/ijms27198726

**Published:** 2026-09-29

**Authors:** Shaopeng Chen, Yuxuan Cheng, Qianqian Zhuang, Yujie Zhang

**Affiliations:** College of Agriculture, Jilin Agricultural Science and Technology College, Jilin 132101, China; chenshaopeng@jlnku.edu.cn (S.C.);

**Keywords:** blueberry, epicuticular wax, multi-omics analysis, WGCNA, triterpenoids, ABC transporter

## Abstract

The cuticular wax (bloom) of blueberries is crucial for postharvest storability and commercial value, yet the regulatory networks underlying its formation remain elusive. Here, we integrated scanning electron microscopy (SEM) with transcriptomic and compartment-resolved metabolomic analyses to elucidate the dynamic establishment of epicuticular wax in blueberry ‘Bluecrop’. SEM revealed an ordered transition of wax crystals from a sparse fibrous structure at the green stage to a dense sponge-like three-dimensional network at maturity. Parallel untargeted liquid chromatography–tandem mass spectrometry (LC-MS/MS) metabolomics of the whole fruit (intracellular precursor pool) and epicuticular wax (extracellular assembly layer) captured the spatiotemporal surge of key triterpenoids, such as oleanolic and ursolic acids, the major constituents associated with wax crystal coarsening. Through weighted gene co-expression network analysis (WGCNA) and LASSO machine learning, we identified co-expression modules, most notably Turquoise, that were closely associated with lipid and triterpenoid synthesis. Hub genes, including a putative *VcMYB96* transcription factor and plasma membrane-localized *VcABCG*-like transporters, are hypothesized to mediate carbon flux reallocation and transmembrane export of hydrophobic precursors. This study provides a “phenotype–transcriptome–metabolome” framework and candidate targets for functional validation and molecular breeding of blueberry cultivars with superior bloom traits.

## 1. Introduction

The plant cuticle is a continuous hydrophobic barrier covering the outer surface of epidermal cells in the aerial parts of higher plants and plays an essential role in plant adaptation to terrestrial environments. As the first line of defense against external environmental stresses, the cuticle consists of a cutin polymer matrix and cuticular wax. It performs crucial physiological functions, including limiting non-stomatal water loss, protecting against pathogen and pest invasion, and shielding tissues from ultraviolet (UV) radiation [1,2].

In blueberries (*Vaccinium* spp.), the fruit surface is covered by a characteristic grayish-white epicuticular wax layer, commonly referred to as “bloom.” This layer not only contributes to important visual attributes, such as surface lightness and color saturation, but also represents a key determinant of fruit commercial quality [3]. Previous studies have demonstrated that bloom integrity is positively correlated with firmness retention capacity and negatively correlated with postharvest water loss. Mechanical damage, rain wash, or removal of the bloom accelerates water transpiration and fruit softening while increasing susceptibility to postharvest diseases [4,5].

From a micromorphological perspective, blueberry epicuticular wax exhibits a highly ordered self-assembled crystalline architecture. Scanning electron microscopy (SEM) studies have demonstrated that blueberry bloom is primarily composed of densely arranged tubule- or rodlet-shaped crystals overlaying an amorphous wax layer [6,7]. Through light scattering effects, these microstructures generate the characteristic glaucous appearance of blueberry fruits and strongly influence surface glossiness and lightness [3]. Furthermore, visible light reflectance properties are closely associated with the density and morphology of specific wax crystals [3]. Crucially, this structural contribution to fruit appearance is largely independent of anthocyanin accumulation, highlighting the physical basis of blueberry visual quality.

At the chemical level, blueberry cuticular wax presents a metabolic composition distinct from that of model species. Unlike *Arabidopsis*, whose waxes are dominated by alkanes and primary alcohols, blueberry epicuticular wax contains exceptionally high levels of triterpenoids, which typically account for more than 50% of the total wax content, with oleanolic acid and ursolic acid as the core components [6,8]. Earlier studies suggested that minor amounts of β-diketones were primarily responsible for the characteristic glaucous appearance of blueberries [3,9]. However, accumulating evidence from chemical ecology and structural biology indicates that triterpenoids and very-long-chain fatty acid (VLCFA) derivatives play central roles in establishing the dense three-dimensional crystalline network of the fruit surface and its associated water-barrier properties. Triterpenoids were also identified as crucial structural components involved in maintaining postharvest water homeostasis and delaying fruit softening [10]. Meanwhile, VLCFAs serve as common precursors for the biosynthesis of various aliphatic wax components, including primary alcohols, aldehydes, and alkanes. Their metabolic flux and abundance directly influence the hydrophobicity and structural compactness of wax crystals [11,12]. Consequently, the coordinated accumulation of triterpenoids and VLCFA-derived compounds constitutes an important metabolic basis for bloom formation in blueberries.

Although the fundamental pathways of plant wax biosynthesis, including fatty acid elongation and the downstream acyl-reduction (alcohol-forming) and decarbonylation (alkane-forming) pathways, have been extensively characterized in *Arabidopsis* and tomato [13,14], the molecular regulatory mechanisms governing wax formation in blueberries remain poorly understood, particularly given their unusually high triterpenoid accumulation. Current transcriptomic studies have identified several candidate genes potentially associated with wax biosynthesis. For example, the expression of the FatB gene, encoding an acyl–acyl carrier protein (acyl-ACP) thioesterase, is highly correlated with wax accumulation in bloom-deficient blueberry mutants identified through bulked segregant analysis (BSA) [15]. In wild bilberry (*Vaccinium myrtillus*), genes involved in VLCFA elongation (*CER26*-like), primary alcohol synthesis (*FAR2*), and alkane synthesis (*CER3*-like) are specifically expressed in the fruit peel during periods of active wax crystal formation [16]. In addition, recent studies have shown that phytohormones such as abscisic acid (ABA) can regulate wax composition and crystal morphology during fruit ripening by modulating the expression of structural genes like *KCS*, *KCR*, and *CER1* [17].

Despite these advances, most previous studies have focused on single-omics analyses or individual candidate genes, lacking a systematic framework integrating phenotypic, metabolic, and transcriptional regulation. In particular, during blueberry fruit ripening, the coordinated regulation of triterpenoid biosynthesis and VLCFA elongation by transcription factors, as well as their contributions to the formation of the dense sponge-like wax crystal network, remain largely unclear. Therefore, in the present study, we investigated bloom formation in blueberry fruit by integrating SEM-based phenotypic analysis, untargeted metabolomics (including parallel profiling of both epicuticular wax and whole fruit), and high-throughput transcriptomics. By constructing a multilayer regulatory network using weighted gene co-expression network analysis (WGCNA), this study aimed to identify key regulatory modules and hub genes associated with blueberry bloom formation. The findings provide novel insights into the molecular mechanisms underlying plant surface wax diversity and offer valuable theoretical and genetic resources for breeding blueberry cultivars with improved storability and enhanced visual quality.

## 2. Results

### 2.1. Microstructural Evolution of Epicuticular Wax During Fruit Development

In the 5000× SEM images of blueberry epidermis at the green fruit stage (Figure 1a), the epicuticular wax consisted of randomly distributed and interconnected fibrous structures, forming a loose network with large pores. Although the majority of the epidermal surface was covered by wax, a relatively smooth underlying basal surface remained visible in certain areas and within the pores. Overall, the wax was relatively uniformly distributed but structurally loose. Quantitative analysis of the 10,000× SEM images (Figure 1b) revealed that wax coverage was 44.96% and surface porosity was 55.04% at the green stage. The fibrous wax structures had an average diameter of 0.49 μm (Figure 1c).

At the veraison stage, when secondary metabolism became highly active, the cuticular wax structure began to undergo a significant transition (Figure 1d). The original fibrous network gradually disintegrated, and the microscopic field showed a mixed morphology characterized by the coexistence of fibrous, irregular granular, and newly formed platelet-like structures. A prominent feature of this transitional phase was the high heterogeneity of the wax coverage, with some regions even displaying noticeable structural discontinuities or wax absence. Although wax coverage (45.40%) and porosity (54.60%) at veraison did not differ substantially from those at the green stage (Figure 1e), the distribution range of crystal diameters widened considerably (Figure 1f). The average diameter slightly increased to 0.54 μm, and this non-uniform size distribution indicated ongoing alterations in wax chemical composition.

At the fully mature stage, the epicuticular wax ultimately self-assembled into a dense, continuous, sponge-like 3D network (Figure 1g). At this point, platelet-like crystals had become the dominant morphological type. These crystals were densely stacked and fused, completely covering the epidermal cells, with no exposed areas visible in the field of view. Quantitative analysis demonstrated that the structural integrity of the wax layer was markedly enhanced at maturity (Figure 1h,i). Wax coverage increased to 61.65%, while surface porosity significantly decreased to 38.35%. Notably, wax crystals underwent pronounced coarsening at this stage, with the average diameter increasing to 0.89 μm. These results indicate the final establishment of the physical barrier structure of the blueberry bloom. Consistent with these morphological changes, gravimetric monitoring of the cotton swabs used for wax collection provided an approximate estimate of the recoverable wax mass, which amounted to 17–122, 20–60, and 60–110 μg per fruit at the green, veraison, and mature stages, respectively, supporting the progressive accumulation of surface bloom during ripening.

### 2.2. Epicuticular Wax Metabolomics Analysis

#### 2.2.1. Multivariate Statistical Analysis of the Epicuticular Wax Metabolome

To obtain an unsupervised overview of the metabolomic data, principal component analysis (PCA) was first performed on the epicuticular wax metabolomic samples. Figure 2 illustrates the spatial distribution of samples across the three developmental stages, with the first and second principal components (PC1 and PC2) explaining 16.70% and 10.63% of the total variance, respectively. Samples from different developmental stages were clearly separated. A subsequent supervised orthogonal partial least squares discriminant analysis (OPLS-DA) model exhibited high explanatory and predictive ability (R2Y = 0.949, Q2 = 0.679), and response permutation testing (*n* = 200) confirmed model validity (permutation *p* < 0.05). The samples followed a distinct developmental trajectory from the green stage (B1) through the veraison stage (B2) to the mature stage (B3). These results indicate that blueberry epicuticular wax undergoes extensive metabolic reprogramming during fruit development, resulting in stage-specific metabolic profiles. In addition, all quality control (QC) samples clustered tightly with minimal dispersion, confirming the reliability and reproducibility of the metabolomic data for subsequent differential metabolite analysis.

#### 2.2.2. Annotation of Secondary Metabolites in Epicuticular Wax

Secondary metabolite annotation revealed that 4686 metabolites were successfully assigned to SuperClass categories in the Human Metabolome Database (HMDB) (Figure 3). At the superclass level, these metabolites were mainly enriched in “Lipids and lipid-like molecules”. This category encompasses many components closely associated with bloom structural assembly, including fatty acyls, prenol lipids, and derivatives of pentacyclic triterpenoids and sesquiterpenoids. Simultaneously, a substantial number of Phenylpropanoids and polyketides were annotated, indicating active secondary metabolism during blueberry fruit development.

#### 2.2.3. Differential Metabolite Analysis of the Epicuticular Wax

As shown in Figure 4, pairwise comparisons of the epicuticular wax metabolomes were performed among the three developmental stages. The results revealed that the differential metabolites (DMs) exhibited pronounced stage-specific patterns. During the transition from the green stage to the veraison stage (B2 vs. B1), 285 upregulated and 218 downregulated metabolites were identified. Among the upregulated metabolites, triterpenoids, including oleanolic acid and ursolic acid, and long-chain fatty acid derivatives were significantly enriched. This metabolic pattern was consistent with the rapid accumulation and structural remodeling of bloom during this stage. As fruit development progressed from veraison to maturity (B3 vs. B2), the metabolic profile shifted markedly. In this comparison, 435 downregulated metabolites were detected, substantially exceeding the 198 upregulated metabolites. A similar trend was observed in the comparison between the mature and green stages (B3 vs. B1), with 135 upregulated and 282 downregulated metabolites identified. These results suggest that epicuticular wax metabolism is highly dynamic during blueberry fruit development, with active metabolite accumulation and remodeling during the early-to-middle transition followed by broader metabolic adjustment at maturity.

#### 2.2.4. KEGG Pathway Enrichment Analysis of the Epicuticular Wax Metabolome

Based on the Kyoto Encyclopedia of Genes and Genomes (KEGG) pathway enrichment analysis (Figure 5), blueberry epicuticular wax formation was primarily associated with lipid metabolism and secondary metabolic pathways. Within lipid metabolism, “glycerophospholipid metabolism” and “cutin, suberine and wax biosynthesis” were significantly enriched. Notably, as fruit development progressed, particularly from veraison to maturity, pathways related to fatty acid elongation and unsaturated fatty acid biosynthesis showed increased metabolic activity. This indicates that the developing fruit enhances the supply of VLCFA precursors and lipid backbones, thereby providing the material basis for epicuticular wax crystal formation. In addition, pathways associated with secondary metabolism, including “flavonoid biosynthesis” and “biosynthesis of phenylpropanoids”, were enriched. These results suggest that during the critical period of bloom formation, blueberry fruit not only develops a hydrophobic wax barrier but also activates secondary metabolite accumulation. Triterpenoids and anthocyanin-related compounds may contribute to the chemical composition and microcrystalline phenotype of blueberry bloom, potentially through coordinated transport and deposition with wax components.

**Figure 3:**
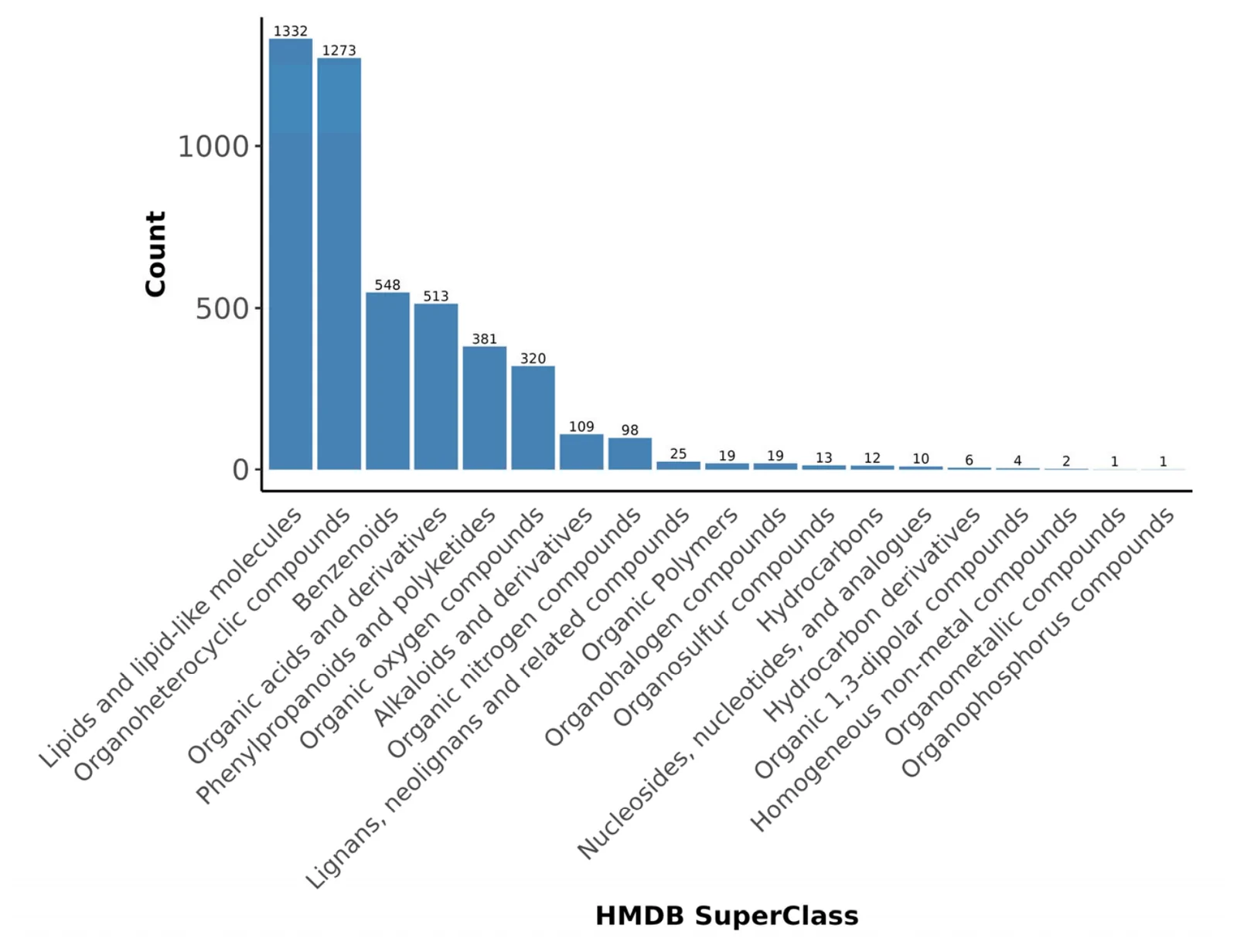
HMDB SuperClass annotation of secondary metabolites in blueberry epicuticular wax.

**Figure 4:**
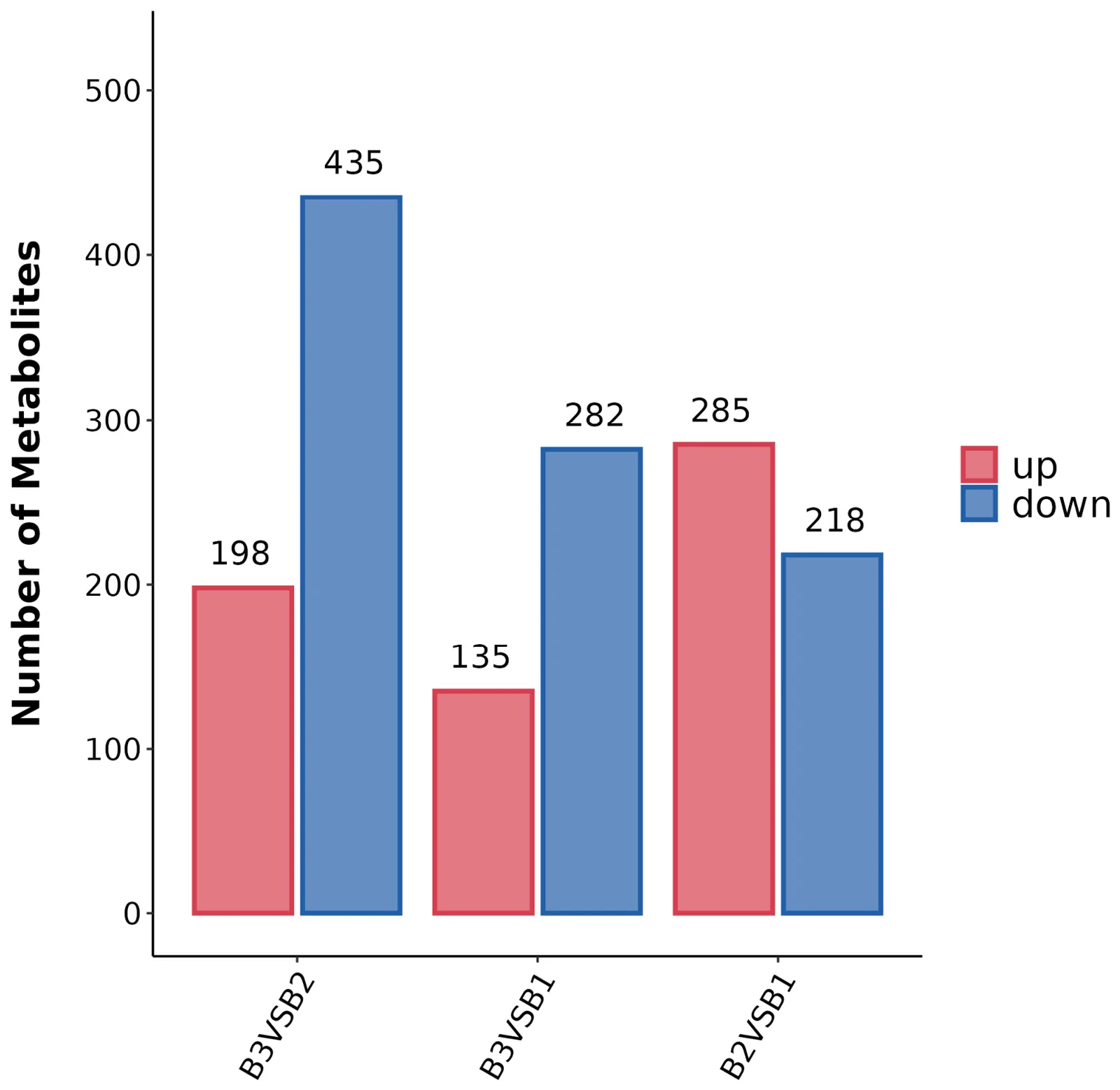
Pairwise differential metabolite analysis of epicuticular wax across developmental stages. Note: Differential metabolites were identified using variable importance in projection (VIP) > 1 from the OPLS-DA model combined with *p* < 0.05.

**Figure 5:**
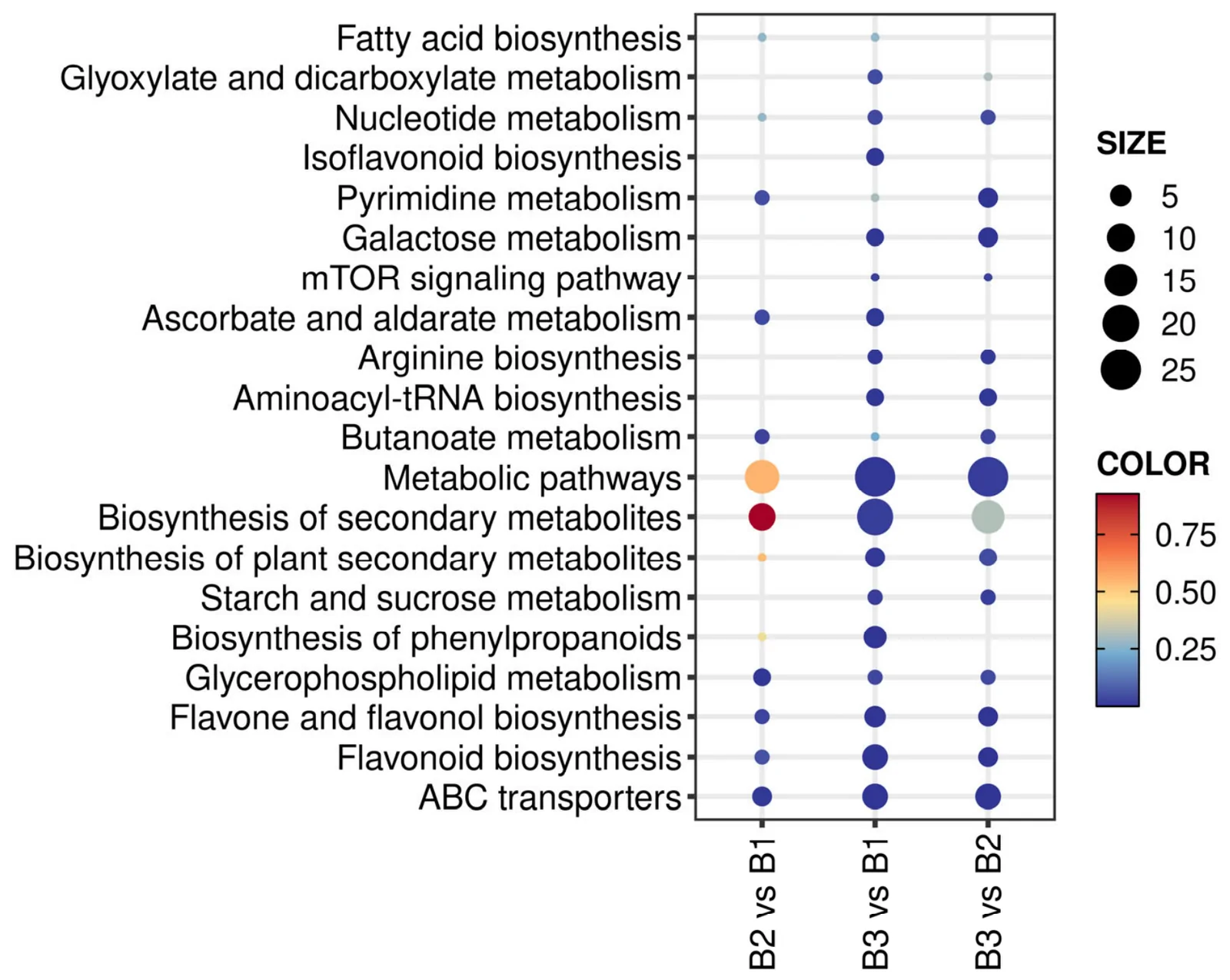
KEGG pathway enrichment analysis of the epicuticular wax metabolome. Note: Bubble size and color encode the number of differential metabolites involved in each pathway and the enrichment significance, respectively.

#### 2.2.5. GSEA Enrichment Analysis of Key Metabolic Pathways

To further explore global changes in metabolic networks in blueberry epicuticular wax from the green stage (B1) to the mature stage (B3), Gene Set Enrichment Analysis (GSEA) was performed on the metabolomic data. Figure 6 presents the Enrichment Plots for four key metabolic pathways in the B3 vs. B1 comparison. The enrichment patterns differed among pathways. “ABC transporters” (Figure 6a) and “galactose metabolism” (Figure 6c) showed positive enrichment, with enrichment peaks positioned on the left side of the ranked list, corresponding to the B3 group. ABC transporters passed the significance threshold (*p* < 0.05), whereas galactose metabolism showed a marginal nominal *p* value (*p* = 0.059), suggesting that metabolites in these pathways tended to be more abundant at the mature stage. In contrast, “glycerophospholipid metabolism” (Figure 6b) and “flavonoid biosynthesis” (Figure 6d) displayed negative enrichment. Their enrichment score curves extended toward negative values, with peaks located on the right side of the ranked list, corresponding to the B1 group. Both pathways were significantly enriched (*p* < 0.01), indicating that metabolites associated with these pathways were predominantly enriched at the green stage (B1) and relatively reduced at maturity.

### 2.3. Whole-Fruit Metabolomics Analysis

The formation of the epidermal cuticle and wax crystals largely depends on the extracellular transport and subsequent self-assembly of lipid precursors synthesized within fruit tissues. Therefore, to elucidate the material sources and metabolic fluxes associated with blueberry bloom development, untargeted metabolomic profiling was further performed on whole-fruit tissues, including both flesh and epidermal cells, following the analysis of surface epicuticular wax metabolites. This approach enabled characterization of the dynamic changes in the intracellular synthesis precursor pool that underpins bloom formation.

#### 2.3.1. Multivariate Statistical Analysis of the Whole-Fruit Metabolome

As shown in the PCA score plot (Figure 7), whole-fruit metabolomic samples from the three developmental stages were clearly separated, with no obvious overlap among groups, indicating stage-specific metabolic characteristics. The samples displayed an ordered developmental trajectory along the positive direction of the PC1 axis, progressing from the green stage (Cm1) through the veraison stage (Cm2) to the mature stage (Cm3). This pattern suggests that blueberry fruit underwent extensive and directional metabolic reprogramming during ripening. Furthermore, all QC samples clustered tightly in the plot, demonstrating the reliability and reproducibility of the untargeted metabolomic data (supervised OPLS-DA model: R2Y = 0.973, Q2 = 0.856; response permutation test, *p* < 0.05) and providing a robust basis for subsequent differential metabolite analysis.

#### 2.3.2. Annotation of Secondary Metabolites in Whole Fruit

Based on the HMDB database, all identified metabolites were classified and statistically analyzed at the SuperClass level. As illustrated in Figure 8, a total of 2753 metabolites were identified in the whole-fruit metabolome and classified into 16 major categories. Among these, “Lipids and lipid-like molecules” constituted the largest category, containing 806 metabolites, highlighting the central role of lipid metabolism in cell membrane construction, energy storage, and signal transduction during fruit development.

This category was followed by “Organoheterocyclic compounds” and “Benzenoids,” with 559 and 374 annotated metabolites, respectively, reflecting complex secondary metabolic networks and the potential metabolic basis of fruit aroma formation. Additionally, “Organic acids and derivatives” and “Phenylpropanoids and polyketides” were also well represented, with 321 and 265 metabolites. These compounds are important contributors to blueberry flavor and nutritional quality and include key precursors or products related to bioactive antioxidants, such as anthocyanins.

#### 2.3.3. Differential Metabolite Analysis in Whole Fruit

As shown in Figure 9, pairwise comparisons of the whole-fruit metabolomes were performed among the three developmental stages. During the transition from the green stage to the veraison stage (Cm2 vs. Cm1), 83 upregulated and 59 downregulated metabolites were identified, suggesting active metabolic adjustment during early fruit development and the onset of ripening. As fruit development progressed from veraison to maturity (Cm3 vs. Cm2), 67 upregulated and 77 downregulated metabolites were detected, indicating substantial remodeling of metabolic pathways during the ripening stage. The comparison between the mature and green stages showed the most pronounced metabolic differences, with 85 upregulated and 77 downregulated metabolites identified in Cm3 vs. Cm1. These results indicate that blueberry fruit ripening is accompanied by extensive remodeling of metabolic networks.

#### 2.3.4. KEGG Pathway Enrichment Analysis of Bloom-Related Metabolites During Fruit Development

Whole-fruit metabolites associated with bloom formation were selected for KEGG pathway enrichment analysis, and the results were visualized using bubble plots. As shown in Figure 10, “glycerophospholipid metabolism” was significantly enriched in all three pairwise comparisons, indicating continuous remodeling of membrane lipid components during fruit development, ripening, and senescence. As fruit entered the veraison and mature stages, significant enrichment of “starch and sucrose metabolism” and “galactose metabolism” suggested active carbon mobilization and cell wall polysaccharide metabolism. These processes may contribute to fruit softening but also provide carbon skeletons and energy for the biosynthesis of epicuticular wax components, particularly VLCFAs and triterpenoid backbones. Notably, differential metabolites involved in “flavonoid biosynthesis” and “biosynthesis of phenylpropanoids” also changed significantly, with enrichment of the “ABC transporters” pathway. These results suggest that during ripening, blueberry fruit not only accumulates quality-related secondary metabolites, such as anthocyanins, but also establishes transport-related processes that may contribute to metabolite compartmentalization and epidermal deposition. Together, these metabolic changes are likely involved in determining the final coloration, nutritional quality, and bloom-associated surface characteristics of mature blueberry fruit.

#### 2.3.5. GSEA Enrichment Analysis of Bloom-Related Metabolic Pathways During Fruit Development

To explore global trends in metabolic networks from the green stage (Cm1) to the mature stage (Cm3), Gene Set Enrichment Analysis (GSEA) was performed on the whole-fruit metabolomic data across developmental stages. Figure 11 presents enrichment plots for four key metabolic pathways across various comparison groups.

As illustrated in Figure 11, these pathways exhibited significant stage-specific enrichment patterns. During the early developmental transition from the green stage to veraison (Cm2 vs. Cm1), “degradation of aromatic compounds” (Figure 11a) and “biosynthesis of plant secondary metabolites” (Figure 11c) showed positive enrichment, with peaks positioned on the left side of the ranked list. Normalized Enrichment Score (NES) was 1.43 (*p* < 0.05) for the degradation of aromatic compounds and 1.38 (*p* = 0.057) for biosynthesis of plant secondary metabolites, indicating that metabolites associated with these pathways were generally more abundant at the veraison stage. At the mature stage, the “biosynthesis of phenylpropanoids” (Figure 11b) and “biosynthesis of unsaturated fatty acids” (Figure 11d) displayed negative enrichment. In the Cm3 vs. Cm1 comparison, the peak of the phenylpropanoid biosynthesis pathway was located on the right side of the ranked list, with an NES value of −1.69 (*p* < 0.01). Similarly, in the Cm3 vs. Cm2 comparison, the biosynthesis of unsaturated fatty acids pathway had an NES of −1.63 (*p* < 0.01), with its peak also located on the right side. These results indicate that metabolites in these pathways were significantly less abundant at the mature stage than at earlier developmental stages.

### 2.4. Transcriptomic Analysis of Fruits

#### 2.4.1. Sequencing Quality Assessment and Screening of Differentially Expressed Genes

To evaluate sequencing reliability and characterize gene expression patterns during fruit development, PCA was first performed. As shown in Figure 12a, biological replicates from the three developmental stages (Ct1, Ct2, and Ct3) exhibited tight intra-group clustering, clear inter-group separation, and a distinct developmental trajectory within the principal component space. These results indicate that developmental stage was the dominant factor driving transcriptomic variation, and that the samples showed good biological reproducibility, providing a reliable basis for subsequent differential expression analyses. The number of differentially expressed genes (DEGs) further revealed the dynamic characteristics of transcriptional regulation during fruit development (Figure 12b). Across the entire developmental period (Ct3 vs. Ct1), 22,285 DEGs were identified, including 4691 upregulated and 17,594 downregulated genes, reflecting the differences in gene expression between mature and young fruits.

Notably, comparisons between adjacent stages displayed an “early burst followed by late stabilization” pattern. During the transition from the green stage to veraison (Ct2 vs. Ct1), 21,342 DEGs were identified, including 4303 upregulated and 17,039 downregulated genes, indicating extensive transcriptional reprogramming at the onset of veraison. In contrast, during the transition from veraison to maturity (Ct3 vs. Ct2), the number of DEGs significantly decreased to 2672, including 1560 upregulated and 1112 downregulated genes. These results demonstrate that transcriptomic remodeling in blueberry fruit occurs mainly during the transition from the green stage to veraison, whereas the gene expression network became relatively stable as the fruit reached full maturity.

#### 2.4.2. GO Functional Enrichment Analysis

To elucidate functional changes in gene expression during blueberry fruit development, Gene Ontology (GO) enrichment analysis was performed for DEGs in each comparison group. Significantly enriched terms related to cuticular wax formation (*p* < 0.05) were selected for visualization. Figure 13 displays 20 representative terms in a bubble chart. In the Ct2 vs. Ct1 comparison, the most significantly enriched GO terms were mainly associated with cellular components and primary metabolic processes. Terms related to the photosystem, such as “chloroplast thylakoid membrane (GO.0009535)” and “photosynthesis (GO.0015979)”, exhibited significant enrichment and contained a large number of DEGs (*p* < 0.05). Furthermore, “fatty acid elongase activity (GO.0009922)”, which is related to wax precursor biosynthesis, was also significantly enriched during this stage. In the Ct3 vs. Ct1 and Ct3 vs. Ct2 comparisons, enrichment patterns shifted as fruit entered the mature stage. Terms such as “wax biosynthetic process (GO.0010025)”, “ABC-type transporter activity (GO.0140359)”, and “lipid localization” displayed high rich factors in the bubble plot. Additionally, genes related to “cuticle development (GO.0042335)”, which is involved in structural establishment of the epidermal barrier, were also significantly enriched at the mature stage.

#### 2.4.3. KEGG Pathway Enrichment Analysis of Differentially Expressed Genes

KEGG pathway enrichment analysis was performed for the DEGs in each comparison group, and significantly enriched pathways related to cuticular wax formation (*p* < 0.05) were selected for visualization. Figure 14 displays 20 representative pathways in a bubble plot. In the Ct2 vs. Ct1 comparison, “cutin, suberine and wax biosynthesis (map00073)” and “fatty acid elongation (map00062)” were significantly enriched, indicating that transcriptional activity related to lipid metabolism was already active during the green-to-veraison transition. Concurrently, the “phenylpropanoid biosynthesis (map00940)” and “plant hormone signal transduction (map04075)” pathways were also enriched and contained numerous DEGs. Across the full developmental comparison, Ct3 vs. Ct1, “ABC transporters (map02010)” was among the most significantly enriched pathways (*p* < 1.0 × 10^−10^), while “biosynthesis of secondary metabolites (map01110)” and “flavonoid biosynthesis (map00941)” consistently maintained high significance. In the late maturation comparison, Ct3 vs. Ct2, “glycerophospholipid metabolism (map00564)” and “sphingolipid metabolism (map00600)” were also significantly enriched, suggesting specific adjustments in membrane lipid metabolism during fruit maturation.

#### 2.4.4. Dynamic Characteristics of GSEA for Metabolic Networks Related to Cuticular Wax Formation at the Mature Stage

GSEA based on transcriptomic data elucidated significant changes in gene set expression patterns during the transition from veraison (Ct2) to maturity (Ct3) in blueberry fruit. As shown in Figure 15, gene sets related to biosynthesis, transport, and energy metabolism, which are closely associated with cuticular wax formation, exhibited coordinated positive enrichment at the mature stage.

In the lipid biosynthesis and transmembrane transport module (Figure 15a–d), gene sets involved in upstream wax precursor biosynthesis, such as “fatty acid elongation” (NES = 2.21), and downstream pathways, such as “cutin, suberine and wax biosynthesis” (NES = 1.99), were significantly positively enriched (*p* < 0.001). This indicates that genes encoding enzymes involved in VLCFA synthesis and wax modification were highly expressed at the mature stage. Notably, genes encoding ABC transporters responsible for lipid secretion also showed strong positive enrichment at this stage (NES = 2.09, *p* < 0.001), suggesting temporal coordination between wax component biosynthesis and extracellular transport. Furthermore, the positive enrichment of “biosynthesis of unsaturated fatty acids” (NES = 1.63, *p* = 0.003) suggests that fruit cells may adjust the physical properties of cuticular wax components during maturation. In the energy metabolism module (Figure 15e,f), the “citrate cycle” (NES = 1.64, *q* = 0.034) was positively enriched, whereas “oxidative phosphorylation” (NES = −1.26, *p* = 0.0014) was modestly negatively enriched. The upregulation of the citrate cycle suggests that mature epidermal cells sustain the supply of carbon skeletons required for the energy-consuming wax biosynthesis and export, while the concurrent negative enrichment of oxidative phosphorylation and photosynthesis-related gene sets reflects the progressive shift from photosynthetic metabolism toward storage and barrier construction during ripening.

In the developmental timing and functional transition module (Figure 15g–i), several gene sets exhibited significant negative enrichment. The downregulation of genes related to “photosynthesis” (NES = −2.63) and “porphyrin metabolism” (NES = −1.92) marked the decline of chloroplast function and chlorophyll degradation during fruit maturation. Meanwhile, the negative enrichment of “flavonoid biosynthesis” (NES = −1.78) indicates that, compared with the rapid accumulation of anthocyanins at the veraison stage (Ct2), transcriptional regulation at maturity (Ct3) partially shifted toward the construction and reinforcement of the epidermal physical barrier.

### 2.5. Dissection of Key Gene Networks Involved in Cuticular Wax Formation Based on Multi-Omics Integrative Analysis

#### 2.5.1. WGCNA Module Identification and Trait Correlation Analysis

To systematically elucidate the relationships among transcriptional changes, blueberry cuticular wax phenotypes, and metabolite remodeling, a weighted gene co-expression network was constructed using transcriptomic, metabolomic, and wax micromorphological data. As shown in Figure 16a, module-trait relationship analysis indicated that different co-expressed gene modules exhibited specific associations with blueberry developmental stages and wax-related traits. Notably, eigengenes of the Turquoise and Blue modules were significantly positively correlated with the mature stage (Ct3) wax phenotype and the accumulation of key lipid metabolites, suggesting that these two modules may play important roles in fruit maturation and epidermal development.

To evaluate the reliability of the network structure within the selected modules, scatter plots of gene significance (GS) versus module membership (MM) were analyzed. The results revealed a strong positive linear correlation between GS and MM for genes in the Turquoise and Blue modules (Figure 16b; Turquoise: cor = 0.706, *p* < 1 × 10^−200^; Blue: cor = 0.865). This indicates that the highly connected genes located at the core of these modules were consistent with the developmental trajectory of wax-related phenotypes.

#### 2.5.2. Biological Functional Annotation of Key Co-Expressed Modules

GO enrichment analysis was performed for genes in the Turquoise and Blue modules to explore their biological functions. The GO enrichment bubble plots showed functional differentiation between these two co-expression networks.

The Turquoise module was primarily enriched in cellular components and processes related to lipid metabolism, including “lipid droplet” and “lipid storage”, “triterpenoid biosynthetic process” and “β-amyrin synthase activity” (Figure 16c). These results were consistent with the abundant accumulation of key triterpenoids, such as oleanolic acid and ursolic acid, observed in the metabolomics data. This suggests that the Turquoise module may act as a regulatory center mediating the biosynthesis of characteristic lipids and triterpenoid components in blueberry cuticular wax.

In contrast, genes in the Blue module were significantly enriched in the “flavonoid biosynthetic process”, “anthocyanin-containing compound biosynthetic process”, and “cinnamic acid biosynthetic process” (Figure 16d). These results suggest that, during fruit development, the biosynthesis of water-soluble secondary metabolites and the formation of the hydrophobic epidermal wax barrier may be regulated by relatively independent yet spatiotemporally coordinated gene network modules.

#### 2.5.3. Identification of Key Driving Genes Based on the LASSO Dimensionality Reduction Model

Co-expression modules typically contain hundreds to thousands of genes. To reduce network redundancy and identify targets with phenotypic predictive value, LASSO regression was applied to candidate hub genes with high module connectivity (MM > 0.90) within the key modules. After dimensionality reduction and feature selection, a set of core regulatory targets closely associated with cuticular wax development was identified. Gene annotation showed that these core genes were mainly associated with the cell membrane system and transmembrane transport functions. For example, VaccDscaff29-processed-gene-291.1 (MM = 0.906) was annotated as a plasma membrane-localized xenobiotic transmembrane transporter/antiporter (GO.0042910) involved in export across the plasma membrane. Similarly, VaccDscaff8-augustus-gene-344.29 (MM = 0.910) was also predicted to be localized to the plasma membrane (GO:0005886). In addition to transmembrane transporters, the LASSO model identified several transcription factors located at higher regulatory levels within the network. For instance, VaccDscaff1-processed-gene-82.0, annotated as a *VcMYB96* homolog, showed high module connectivity (MM = 0.971). In model plants such as *Arabidopsis*, *MYB96* has been reported to regulate VLCFA elongation and wax biosynthesis, suggesting that this gene might play a key regulatory role in wax crystal coarsening and densification in blueberry.

Combined with module functional annotation, these results suggest that the Turquoise module may mediate the biosynthesis of wax lipid and triterpenoid precursors in blueberry epidermal cells. Subsequently, high-connectivity plasma membrane transporters may participate in the extracellular export of hydrophobic substances, thereby promoting the formation of the dense wax crystal structures. Supplementary Table S2 summarizes the key candidate genes involved in blueberry cuticular wax formation identified through this analysis, along with their topological characteristics and putative functions.

By integrating WGCNA with the LASSO penalized regression model, we reduced network redundancy and established a data-driven predictive model. This dual-machine-learning approach pinpointed the most critical regulatory hubs (putative *VcMYB96* and *VcABCG*-like transporters). This stringent mathematical dimensionality reduction minimizes false positives and provides a prioritized target library for understanding the compartment-specific reallocation of triterpenoids and lipid precursors during fruit ripening (Supplementary Table S2).

### 2.6. Expression Profiling of Core Regulatory Genes Associated with Wax Crystal Self-Assembly

To validate the potential biological roles of the core genes identified by the WGCNA and dimensionality reduction models, quantitative expression analysis was performed for hub genes associated with the wax metabolism network. These genes were selected to evaluate their possible involvement in the self-assembly of blueberry cuticular wax crystals and the establishment of the epidermal physical barrier.

Structurally intact cuticular wax crystals are not directly shaped by cells but are formed through extracellular phase transition and self-assembly following the accumulation of hydrophobic metabolites. Quantitative analysis showed that genes encoding putative rate-limiting enzymes involved in triterpenoid and VLCFA biosynthesis, including *VcBAS*-like and *VcKCS*-like, were upregulated from the veraison stage and reached peak expression at maturity. Similarly, the plasma membrane-localized transporter *VcABCG*-like and the auxiliary secretory lipid transfer protein *VcLTPG*-like also reached peak expression at the mature stage (Table S3).

This coordinated expression pattern suggests temporal synchronization between lipid biosynthesis and transmembrane transport. Such coordination may promote the efficient export of endoplasmic reticulum-derived hydrophobic lipid precursors across the plasma membrane and cell wall, leading to extracellular accumulation of wax monomers. This accumulation may further facilitate their self-assembly into a continuous lamellar and sponge-like crystalline network on the fruit surface.

Further network analysis indicated that the high-connectivity transcription factors *VcMYB96*-like and *VcWIN1/SHN1*-like may function as regulatory hubs in this process. Their elevated expression may activate downstream carbon-chain elongation pathways and promote the expression or activity of plasma membrane transporter complexes, thereby linking intracellular biosynthesis with extracellular secretion.

Because a continuous and dense cuticular wax layer contributes to the characteristic bloom appearance of blueberry fruit and serves as a crucial physical barrier against postharvest water loss, the expression levels of these core genes were closely correlated with wax crystal coverage and morphological development. Therefore, hub genes such as *VcMYB96*-like and *VcABCG*-like may serve as potential molecular markers for marker-assisted breeding aimed at improving blueberry bloom formation, storability, and postharvest quality (Supplementary Table S3).

## 3. Discussion

### 3.1. Spatiotemporal Synergy Between Cuticular Wax Micro-Morphological Changes and the Water Barrier Function

The microcrystal morphology of plant cuticular wax plays a fundamental role in determining its physical barrier function, optical properties, and contribution to postharvest fruit quality [1,2]. SEM observations showed that blueberry cuticular wax formation exhibited dynamic and ordered self-assembly characteristics. The wax microstructure gradually changed from a loose fibrous network at the early developmental stage to a continuous sponge-like three-dimensional network dominated by platelet-like crystals at the fully mature stage. This morphological trajectory is consistent with the general physical principles of wax crystallization observed in model plants and may also reflect a specialized surface structure developed by blueberry fruit to adapt to environmental stress [18,19].

Wax micromorphology is the visible outcome of the spatial remodeling and phase transitions of the underlying chemical components. Combined with high-throughput metabolomic analysis, the results showed that specific triterpenoids, including oleanolic acid, ursolic acid, and their derivatives, along with other lipids, were significantly enriched in mature fruits. Previous studies on blueberry peel structure and chemical ecology have shown that high proportions of triterpenoids and β-diketones are key molecular components involved in the transition from an amorphous matrix to highly ordered tubular or platelet-like crystals [6,9]. In the present study, extensive metabolic network remodeling from the green stage to veraison (Figure 4 and Figure 9), together with significant enrichment of the fatty acid elongation pathway (Figure 5), likely provided abundant VLCFA precursors for the self-assembly of complex crystal structures at late developmental stages.

Importantly, the dense sponge-like crystal network enriched in triterpenoids may confer multiple biophysical advantages. Recent studies have shown that a dense cuticular wax layer can enhance visible light scattering on the fruit surface, producing the characteristic blueberry bloom independently of anthocyanins and thereby influencing commercial value [3]. Moreover, a continuous three-dimensional wax network may increase the tortuosity of water diffusion across the epidermis, thereby reducing cuticular transpiration [12]. Postharvest water loss in blueberries occurs through the stem scar and cuticle, and triterpenoid-rich waxes help maintain water homeostasis during storage [5,10]. Therefore, the dense wax crystal structure established at maturity, as observed in this study, may be important for delaying fruit softening and enhancing resistance to postharvest pathogen infection.

### 3.2. Metabolic Network Remodeling and Hormonal Cross-Regulation of Wax and Secondary Metabolism

Fruit ripening is generally accompanied by extensive redirection of intracellular metabolic fluxes, resource reallocation, and complex endogenous hormonal signaling [20,21]. Multi-omics integrative analysis revealed potential intersections and coordinated interactions between primary lipid metabolism and secondary metabolic pathways, including flavonoid and triterpenoid biosynthesis, during blueberry fruit development. GSEA indicated that, during the early developmental stages, metabolic activity was mainly associated with glycerophospholipid metabolism and the construction of the basal membrane system. As blueberry fruits entered the mature stage, metabolic flux shifted toward pathways related to “ABC transporters” and wax precursor biosynthesis (Figure 15).

By integrating whole-fruit and epicuticular wax metabolomes in parallel, this study clarified the spatial logic of cuticular wax biosynthesis. Here, the term “compartment-resolved” refers specifically to the parallel profiling of two chemically distinct fruit compartments, namely the extracellular epicuticular wax layer and the intracellular whole-fruit precursor pool, rather than to imaging-based spatial mapping of metabolites within tissues. The whole-fruit metabolome reflected dynamic changes in the intracellular precursor pool within the endoplasmic reticulum and cytosol, whereas the epicuticular wax metabolome represented the outcome of extracellular assembly on the fruit surface after lipid precursors were secreted and modified. The material flow and spatial communication between these metabolic pools may be mediated by transmembrane transport systems, particularly ABC transporters, which were significantly enriched in the transcriptome data.

At maturity, blueberry fruits showed both anthocyanin accumulation and active cuticular wax biosynthesis, suggesting finely regulated carbon and energy allocation. The functional differentiation between the Turquoise module, enriched in lipid and triterpenoid biosynthesis genes, and the Blue module, enriched in flavonoid biosynthesis genes, further supported the spatiotemporal coordination of these two metabolic branches at the transcriptomic level (Figure 16) [22]. Recent transcriptome-metabolome association studies have also indicated that plants can balance multiple carbon fluxes through coordinated gene networks [23,24]. This metabolic remodeling is also closely regulated by endogenous hormones. Recent studies have shown that abscisic acid (ABA) plays an important role in blueberry fruit ripening and anthocyanin accumulation, promoting VLCFA elongation and triterpenoid wax biosynthesis, and increasing the accumulation of oleanolic acid and ursolic acid in blueberry peel by upregulating members of the *OSC* and *CYP716A* gene families [17,20].

With respect to the terminal steps of the triterpenoid biosynthetic pathway, it should be noted that the current blueberry genome annotation, which relies primarily on homology to related Ericaceae species, does not yet provide complete coverage of the scaffold oxidation steps: no *CYP716A*-family homologs were recovered in the present annotation, and candidate oxidosqualene cyclase-type genes were annotated as β-amyrin/lupeol synthase-like sequences. Pathway-level resolution of triterpenoid scaffold oxidation therefore remains incomplete in the current dataset, and *BAS*-type candidates were accordingly prioritized for quantitative real-time PCR (qRT-PCR) validation (Table S3). Likewise, interpretations involving ABA-associated regulation (e.g., *NCED*, *PP2C*, and *SnRK2* family members) are based on transcript abundance patterns and literature precedents; ABA contents were not directly measured in this study, and the corresponding hormonal evidence should be regarded as inferential.

In addition, exogenous signaling molecules such as melatonin have also been reported to improve blueberry storability by altering cuticular wax components [25]. Together with the positive enrichment of the citrate cycle at maturity observed in this study (Figure 15f), these findings suggest that blueberry epidermal cells may coordinate mitochondrial respiratory activity with the expression of rate-limiting genes involved in VLCFA synthesis, such as *KCS* members of the *CER* gene family, in response to hormone levels [26,27]. This coordination may provide ATP and carbon skeletons required for energy-intensive secondary metabolism and wax biosynthesis.

### 3.3. Putative Involvement of Transporters in Extracellular Lipid Secretion

After cuticular wax components are synthesized in the endoplasmic reticulum, highly hydrophobic lipid and triterpenoid molecules must cross the plasma membrane, move through the apoplast and cell wall space, and ultimately self-assemble into wax crystals on the outer surface of the cuticle [28]. This export process, linking the intracellular precursor pool with the extracellular wax assembly layer, depends on specific transmembrane transport systems [29,30].

By integrating WGCNA topology with LASSO-based dimensionality reduction, this study identified several candidate genes that may participate in transmembrane export of wax-related compounds. For example, VaccDscaff29-processed-gene-291.1, a putative *VcABCG11* homolog (MM = 0.906), and VaccDscaff8-augustus-gene-344.29, a putative *VcABCG12*/*CER5* homolog (MM = 0.910), were annotated as plasma membrane-localized transmembrane transporters/antiporters. These genes may be involved in the export of lipid and triterpenoid precursors to the cuticle. In addition, the high co-expression of VaccDscaff6-snap-gene-78.41, a putative *VcCER1* homolog (MM = 0.907) with metal ion binding activity, suggests that it may function as an auxiliary component associated with lipid modification or transport-related processes. These candidate genes may contribute to extracellular lipid secretion and influence the deposition and thickness of the cuticular wax [29,31].

Through the integrated multi-omics analysis, several high-weight candidate genes were identified, with both GS and MM values exceeding 0.90. Such “double-high” genes may have strong associations with wax-related phenotypes and are therefore valuable candidates for further functional validation. For example, VaccDscaff1-processed-gene-82.0, a putative *VcMYB96*-like transcription factor candidate (GS = 0.910), exhibited a strong positive association with wax crystal development, including the increase in average crystal diameter from 0.49 μm to 0.89 μm measured by SEM. These highly connected and phenotype-associated genes may represent blueberry-specific regulators of secondary metabolic modification or key transcriptional components of the wax biosynthesis network. Overall, by applying a phenotype-metabolome-transcriptome systems biology framework, this study provides new insight into the molecular basis of blueberry cuticular wax morphogenesis. The identified candidate genes also offer a valuable resource for future functional validation, as well as for marker-assisted breeding of blueberry cultivars with improved bloom appearance, postharvest quality, and superior storability.

### 3.4. Study Limitations and Future Perspectives

While this study integrated transcriptomic and compartment-resolved metabolomic data to delineate the spatiotemporal regulatory network of blueberry cuticular wax, several limitations should be acknowledged. First, due to the current technical bottlenecks in achieving highly efficient and stable genetic transformation in woody fruit crops like blueberries, in vivo functional validation of the identified hub genes was not performed. Nevertheless, our WGCNA-LASSO framework has substantially narrowed the candidate gene pool and provided well-supported targets for future transient expression assays in blueberry or heterologous validation in model plants.

Second, regarding the compartment-resolved metabolomic design, we observed inconsistencies in the number of differential metabolites between the whole-fruit and epicuticular wax datasets. This is consistent with the concept of the “matrix dilution effect”. The whole-fruit extracts represent the intracellular precursor pool, where trace epicuticular wax precursors can be masked by highly abundant primary metabolites (sugars and organic acids), thus justifying our specialized epicuticular wax sampling. Furthermore, while GC-MS is traditionally used for analyzing highly hydrophobic very-long-chain alkanes, previous studies and our current data confirmed that large triterpenoid macromolecules are the dominant functional drivers of blueberry bloom. The untargeted LC-MS/MS platform utilized in this study is well suited for the detection of these triterpenoids, thereby accurately capturing the chemical basis of the micro-morphological phase transitions observed via SEM.

Third, the functional implications of the wax architecture were inferred from the morphological and chemical evidence obtained here together with previous reports. In this study, wax recovery was monitored gravimetrically by weighing the cotton swabs before and after wiping, indicating recoverable wax masses on the order of tens of micrograms per fruit. The barrier-related properties attributed to the dense sponge-like crystalline network will be examined directly in follow-up work, which will help test the structure–function relationships proposed here. In addition, the water activity (Aw) of the fruit, which governs the rates of moisture-dependent biochemical reactions (e.g., lipid oxidation) and thus postharvest deterioration, was not measured in this study; its determination, together with the barrier-related measurements described above, is planned in the follow-up work. Fourth, the WGCNA was constructed from nine transcriptomic samples (three developmental stages × three biological replicates), which is below the sample size generally recommended for reliable module detection (*n* ≥ 15); module assignments may therefore partly reflect developmental staging, and the co-expression findings should be verified in larger, independently sampled cohorts. Fifth, as stated in Section 4.3.2 and Section 4.4.4, differential metabolites and DEGs were called using unadjusted *p*-value thresholds; given the genome-wide scale of both datasets, the absence of formal multiple-testing correction may inflate the number of nominal hits, and the associated findings should be interpreted as putative; nevertheless, a sensitivity analysis of the transcriptomic data demonstrated that the principal pathway-level conclusions were retained under FDR correction (*q* < 0.05) combined with a low-expression filter (mean FPKM ≥ 1) (Section 4.3.2). Sixth, in the absence of authentic-standard confirmation for individual triterpenoids, metabolite identifications are regarded as Metabolomics Standards Initiative (MSI) Level 2 putative annotations.

## 4. Materials and Methods

### 4.1. Experimental Design and Sample Collection

The blueberry cultivar ‘Bluecrop’ was cultivated at the horticultural farm of Jilin Agricultural Science and Technology College and used as the experimental material in this study. Sampling was conducted in July 2025 at three developmental stages with distinct phenotypic characteristics: green, veraison, and maturity. A completely randomized design was employed. Samples were divided into three analytical groups. Group 1 was used for SEM-based phenotypic profiling to characterize the dynamic changes in the microcrystalline structure of epicuticular wax. To ensure statistical representativeness, the wax crystal size was determined using ImageJ software (version 1.54f) by randomly measuring the diameter of at least 30 individual crystalline structures within a defined 10 μm × 10 μm area from three independent biological replicates per stage. Group 2 was used for epicuticular wax metabolomics. For this group, the fruit surface was gently wiped with sterile absorbent cotton to collect epicuticular wax from each developmental stage. Fruits at each stage were divided into three bulk lots, each of which was split into two sub-lots for wax collection, yielding six replicate wax samples per stage (18 samples in total). Group 3 was used for whole-fruit transcriptomics and metabolomics analyses. Fruit tissues were collected for transcriptomic analysis (three biological replicates per stage), and six biological replicates were used for metabolomic analysis at each stage. Immediately after collection, all samples for omics analyses were snap-frozen in liquid nitrogen and stored at −80 °C. Samples used for SEM observation were stored at 4 °C. Detailed sample information is provided in Table 1. Notably, Group 2 and Group 3 were jointly designed as a compartment-resolved metabolomic profiling strategy. The whole-fruit samples (Group 3) were utilized to characterize the intracellular precursor pool, whereas the epicuticular wax samples (Group 2) represented the extracellular assembly layer. This parallel design enabled targeted comparison of metabolite abundance between the intracellular synthesis and extracellular deposition compartments, simultaneously accounting for the matrix dilution effect where abundant primary metabolites in whole fruits may mask trace wax precursors.

### 4.2. SEM Analysis

#### 4.2.1. Sample Preparation

Fresh blueberry fruits were selected from each developmental stage. Peel tissue blocks, no larger than 10 mm × 10 mm in area and 5 mm in thickness, were excised from the equatorial region of the fruits using sterile scissors and placed on clean, transparent glass slides. To preserve the native structure of the epicuticular wax, conventional chemical fixation and gradient dehydration were omitted, due to the high-water content of the blueberry peel and the fragile nature of the wax layer. Prior to mounting, free surface moisture was gently removed with absorbent paper to prevent abnormal chamber pressure caused by excessive water evaporation during SEM observation. The sample surfaces were then gently purged with high-purity nitrogen to remove micro-dust and impurities. The prepared samples were mounted on sample stubs using double-sided conductive carbon tape to ensure adequate contact and reduce charging effects.

#### 4.2.2. Microscopic Observation and Image Acquisition/Analysis

The micromorphology of blueberry epicuticular wax was observed using Thermo Fisher QUANTA 200 and Thermo Fisher ChemiAxia scanning electron microscopes (Thermo Fisher Scientific, Waltham, MA, USA). Given that fresh blueberry peel is hydrated and non-conductive, observations were performed in low-vacuum mode to minimize dehydration, shrinkage, and wax structural damage that may occur under high-vacuum conditions. The operating parameters were as follows: accelerating voltage, 20 kV; chamber pressure, 130 Pa; detector, secondary electron detector (SED); and working distance, 5–10 mm. After chamber pressure stabilized within the appropriate range (10–150 Pa), the observation fields were first located at low magnification (50–100×), and then gradually increased to high resolution (>1000×). Focus and stigmation were adjusted until clear images were obtained. After image acquisition, the chamber was returned to atmospheric pressure, and the samples were retrieved. SEM images of blueberry bloom were imported into ImageJ for analysis. After scale calibration, images were converted to grayscale. Contrast was adjusted to enhance crystal visibility, and images were binarized for subsequent analysis. Representative peel regions were selected to manually evaluate and annotate bloom coverage, crystal morphology, and cross-sectional microstructures.

### 4.3. Transcriptome Sequencing and Data Analysis

#### 4.3.1. RNA Extraction, Library Construction, and Sequencing

Frozen fruit samples collected at the green, veraison, and mature stages were ground in liquid nitrogen, and total RNA was extracted using TRIzol reagent according to the manufacturer’s protocol. RNA quantity and purity were assessed using a NanoDrop 2000 spectrophotometer (Thermo Fisher Scientific, Waltham, MA, USA), and RNA integrity was evaluated using an Agilent 2100 Bioanalyzer (Agilent Technologies, Santa Clara, CA, USA). Only samples meeting the quality thresholds of concentration > 50 ng/μL, RNA integrity number (RIN) > 7.0, and total RNA > 1 μg were used for subsequent analyses. Qualified RNA samples were used for cDNA library construction.

Briefly, poly(A) mRNA was enriched using Oligo(dT) magnetic beads and fragmented in a fragmentation buffer. First-strand cDNA was synthesized using random hexamer primers, followed by second-strand cDNA synthesis. Subsequently, end repair, A-tailing, adapter ligation, PCR amplification, and library purification were performed according to the standard protocol, generating strand-specific libraries with insert sizes of 300 bp ± 50 bp. Library construction and sequencing were conducted by LC-Bio Technology Co., Ltd. (Hangzhou, China) using the Illumina NovaSeq 6000 platform (Illumina, Inc., San Diego, CA, USA) with a paired-end 150 bp (PE150) sequencing strategy. Per-sample quality control statistics of the sequencing data are summarized in Table S4. Briefly, each library yielded 36.0–46.1 million raw reads (5.40–6.91 Gb); after filtering, 34.7–44.5 million clean reads (5.21–6.67 Gb) were obtained, with valid read ratios of 96.34–96.62%, Q20 percentages of 100%, Q30 percentages ≥ 99.10%, and GC contents of approximately 46%.

#### 4.3.2. Bioinformatics Analysis

Raw sequencing reads were processed using Cutadapt to remove adapter sequences and low-quality reads, and sequence quality was assessed using FastQC (version 0.10.1), thereby yielding high-quality clean data. The clean reads were aligned to the blueberry reference genome (*Vaccinium corymbosum* cv. ‘Draper’ v1.0) using HISAT2 software (version 2.2.1). Gene expression levels were normalized and calculated as fragments per kilobase of transcript per million mapped reads (FPKM). Differentially expressed genes (DEGs) between developmental stages were identified using the DESeq2 package (version 1.22.2) in R (version 3.6). Genes with |log2(Fold Change)| ≥ 1 and *p* < 0.05 were considered significantly differentially expressed. Given the genome-wide scale of the transcriptomic data, primary DEG calling employed unadjusted *p*-value thresholds, which is acknowledged as a limitation (Section 3.4). A sensitivity analysis was performed to assess the robustness of the DEG results: when *p*-values were corrected for multiple testing using the Benjamini–Hochberg false discovery rate (*q* < 0.05), 18,481, 19,773, and 1020 DEGs were retained for Ct2 vs. Ct1, Ct3 vs. Ct1, and Ct3 vs. Ct2, respectively; applying an additional low-expression filter (mean FPKM ≥ 1) further reduced these numbers to 10,676, 11,328, and 792, while the key pathway-level conclusions remained unchanged. Based on the identified DEGs, Gene Ontology (GO) annotation and Kyoto Encyclopedia of Genes and Genomes (KEGG) pathway enrichment analyses were conducted using the clusterProfiler package in R to identify biological processes and metabolic pathways associated with blueberry bloom formation.

### 4.4. Untargeted Metabolomics Analysis

#### 4.4.1. Sample Preparation and Metabolite Extraction

Two types of sample matrices, namely epicuticular wax and whole fruit, were processed separately prior to metabolomic analysis. For epicuticular wax samples, fresh blueberry fruits from the green, veraison, and mature stages were gently wiped with sterile absorbent cotton until the surface bloom was completely removed, as confirmed visually. Each wax-collection sub-lot comprised 103–210 g of fresh fruits, corresponding to approximately 100–190 fruits (mean fruit diameters of 13.2 ± 0.7, 13.7 ± 1.2, and 14.9 ± 1.7 mm at the green, veraison, and mature stages, respectively; *n* = 36 fruits per stage). The cotton swabs were weighed before and after wiping to gravimetrically monitor the amount of wax recovered, and wiping was performed with uniform stroke intensity until no visible bloom remained, so as to standardize the collected surface area. The cotton containing the adsorbed wax was immediately frozen in liquid nitrogen and stored at −80 °C. For whole-fruit samples, frozen blueberry fruits were ground into fine powder in liquid nitrogen, and 50 mg of homogenized powder was used for metabolite extraction.

Metabolites were extracted using a modified methanol precipitation method. Briefly, 500 μL of pre-chilled 80% aqueous methanol containing internal standards was added to the cotton samples and the fruit powder. After vortex mixing, samples were sonicated in an ice bath for 5–10 min and then incubated at −20 °C for 30 min to precipitate proteins. The mixtures were centrifuged at 20,000× *g* for 10 min at 4 °C, and the supernatants were transferred to autosampler vials for subsequent UPLC-MS/MS analysis. To monitor instrument stability and analytical reproducibility, equal volumes of supernatants from all samples were pooled to prepare quality control (QC) samples.

#### 4.4.2. Chromatographic and Mass Spectrometric Conditions

Chromatographic separation was performed using a Thermo Vanquish Ultra-High Performance Liquid Chromatography (UHPLC) system (Thermo Fisher Scientific, Waltham, MA, USA) coupled with an ACQUITY UPLC HSS T3 column (100 mm × 2.1 mm, 1.8 μm; Waters, Milford, MA, USA). The mobile phase consisted of 0.1% formic acid in water (solvent A) and acetonitrile (solvent B). The gradient elution program was set as follows: 0–1 min, 1% B; 1–8 min, 1–99% B; 8–10 min, 99% B; 10–10.1 min, 99–1% B; and 10.1–12 min, 1% B. The flow rate was maintained at 0.35 mL/min, the column temperature was set at 40 °C, and the injection volume was 4 μL.

Mass spectrometry detection was conducted using a Thermo Q Exactive^TM^ HF-X hybrid quadrupole-Orbitrap mass spectrometer (Thermo Fisher Scientific, Waltham, MA, USA) equipped with an electrospray ionization (ESI) source. Data were acquired in both positive (ESI+) and negative (ESI-) ionization modes using full MS scan and data-dependent MS/MS (dd-MS2) acquisition. The operating parameters were set as follows: sheath gas flow rate, 40 arb; auxiliary gas flow rate, 10 arb; spray voltage, 3.5 kV in positive mode/−2.5 kV in negative mode; capillary temperature, 320 °C; full MS resolution, 60,000; MS/MS resolution, 15,000; and scan range, *m*/*z* 70–1050.

#### 4.4.3. Data Processing and Metabolite Identification

Raw UPLC-MS/MS data were processed using XCMS software for peak detection, peak alignment, retention time correction, and peak area extraction. The resulting data matrix was normalized and subjected to missing value imputation (using the K-nearest neighbors (KNN) algorithm implemented in metaX software). Metabolites were identified based on accurate mass-to-charge ratios (mass error < 10 ppm), isotopic distributions, and MS/MS fragmentation spectra through comparison with the HMDB, KEGG, and in-house standard databases. In the absence of confirmation by co-elution with authentic standards for individual triterpenoids, identifications of oleanolic acid, ursolic acid, and related compounds are reported as putative annotations (Metabolomics Standards Initiative Level 2) based on accurate mass and MS/MS spectral matching. During metabolite identification, special attention was given to large triterpenoid macromolecules, which are the predominant functional drivers of the epicuticular wax bloom in blueberries. The untargeted LC-MS/MS platform utilized in this study provides high sensitivity for the detection and quantification of these triterpenoids compared to traditional GC-MS analysis.

#### 4.4.4. Differential Metabolite Analysis in the Whole Fruit

Principal Component Analysis (PCA) and Orthogonal Partial Least Squares Discriminant Analysis (OPLS-DA) were performed using SIMCA software (Version 14.1, Umetrics, Umeå, Sweden) to evaluate differences in metabolic profiles among developmental stages and identify differential metabolites. Differential metabolites were screened based on Variable Importance in Projection (VIP > 1) from the OPLS-DA model and a *p*-value < 0.05 from Student’s t-test; multiple-testing correction was not applied at the metabolite level, which is acknowledged as a limitation (Section 3.4). Identified metabolites were subsequently mapped to the KEGG database for pathway enrichment analysis. For the transcriptomic data, functional enrichment analysis was performed using Gene Set Enrichment Analysis (GSEA), whereas for the metabolomic data, Over-Representation Analysis (ORA) via the KEGG database was applied, as this method is better suited to small-molecule chemical entities.

### 4.5. WGCNA Construction and Multi-Omics Integrative Analysis

#### 4.5.1. Data Preprocessing and Phenotypic Quantification

To construct a robust co-expression network, transcriptomic data were first filtered to remove lowly expressed genes with FPKM values < 1. For wax micromorphological images obtained by SEM, quantitative image analysis was performed using ImageJ software (NIH, USA). Representative SEM images from each sample group were selected, and wax crystal coverage (%) and crystal density were calculated using threshold segmentation, allowing visual features to be converted into quantitative phenotypic data. Concurrently, the relative abundance data of the differential metabolites identified by metabolomic analysis were integrated to construct a comprehensive phenotype-metabolite trait matrix.

#### 4.5.2. Construction of Weighted Gene Co-Expression Network

To identify gene modules and core targets associated with blueberry bloom development and secondary metabolite accumulation, a weighted gene co-expression network was constructed using the WGCNA package (v1.71) in R. Genes with extremely low expression levels (FPKM < 0.5) were filtered out, and Pearson correlation coefficients were calculated among the remaining genes. A soft-thresholding power was selected to ensure that the network approximated scale-free topology. The adjacency matrix was then transformed into a Topological Overlap Matrix (TOM) to minimize false-positive associations. Co-expressed gene modules were identified using the dynamic tree cut algorithm, with the minimum module size (minModuleSize) set to 30. Correlations between the Module Eigengenes (MEs) and the bloom-related phenotypic parameters, as well as the abundance of characteristic metabolites, were calculated to identify the key responsive modules. Within the target modules, Module Membership (MM) and Gene Significance (GS) were calculated for each gene. Genes meeting the high-connectivity thresholds were defined as the hub genes within their respective modules. GO and KEGG pathway enrichment analyses were performed for all genes in the key modules using the clusterProfiler package in R, with significance assessed using a hypergeometric test (*p* < 0.05). To further identify core regulatory genes, Least Absolute Shrinkage and Selection Operator (LASSO) penalized regression was applied to the preliminarily screened high-connectivity hub genes (MM > 0.90). This approach enabled feature selection and dimensionality reduction, ultimately identifying a core set of candidate genes strongly associated with blueberry bloom development. For the weighted gene co-expression network analysis (WGCNA), although the transcriptomic dataset consisted of 9 samples (3 stages × 3 replicates), the network was constructed as an exploratory screen for candidate modules. The substantial inter-group transcriptomic variance observed during the transition from the green to mature stages, which far exceeds intra-group biological variation, supports the reliability of the module assignments. To further eliminate network redundancy and false positives, low-abundance transcripts (average FPKM < 5) were excluded, and a LASSO machine learning regression model was integrated for secondary dimensionality reduction and cross-validation.

### 4.6. Quantitative Real-Time PCR (qRT-PCR) Analysis

To validate the reliability of the RNA-Seq data and determine the expression patterns of core genes, total RNA was extracted from ground blueberry fruit samples using TRIzol reagent according to the manufacturer’s instructions. RNA concentration and purity were assessed using a NanoDrop 2000 spectrophotometer (Thermo Fisher Scientific, Waltham, MA, USA), and RNA integrity was determined using an Agilent 2100 Bioanalyzer (Agilent Technologies, Santa Clara, CA, USA). High-quality RNA samples were used for first-strand cDNA synthesis using reverse transcriptase according to the manufacturer’s instructions.

Gene-specific primers for the 15 selected hub genes and the internal control gene were designed using Primer Premier 5.0. The blueberry glyceraldehyde-3-phosphate dehydrogenase (GAPDH, CF811166) gene, which exhibits highly stable expression during blueberry fruit development, was utilized as the internal reference for normalization of relative transcript levels (Supplementary Table S1).

The qRT-PCR assays were performed on a LightCycler 480 System (Roche Diagnostics, Mannheim, Germany) or an equivalent Real-Time PCR System using SYBR Green Master Mix. Each 20 μL reaction contained 10 μL of 2× SYBR Green Master Mix, 0.5 μL of each forward and reverse primer, 1 μL of diluted cDNA template, and RNase-free water added to a final volume of 20 μL. The thermal cycling conditions consisted of initial denaturation at 95 °C for 3 min, followed by 40 cycles of 95 °C for 10 s and 60 °C for 1 min. A melting curve analysis was performed at the end of the reaction to confirm amplification specificity. All qRT-PCR reactions were performed with three independent biological replicates and three technical replicates. Relative expression levels of the target genes were calculated using the 2^−ΔΔCt^ method, with the green fruit stage (Ct1) serving as the baseline calibrator.

## 5. Conclusions

In summary, this study constructed a comprehensive “phenotype–transcriptome–metabolome” predictive model to elucidate the spatiotemporal regulatory mechanisms of blueberry cuticular wax formation. By integrating SEM and compartment-resolved metabolomic profiling, we showed that the micro-morphological phase transition of wax crystals is strongly associated with the massive extracellular accumulation of triterpenoids, notably oleanolic and ursolic acids. Furthermore, a dual machine-learning strategy (WGCNA coupled with LASSO) pinpointed the key regulatory modules governing the parallel biosynthesis of flavonoids and triterpenoids. We identified high-weight hub genes, particularly *VcMYB96* and plasma membrane-localized *VcABCG*-like transporters, which putatively serve as candidate key regulators of intracellular carbon flux reallocation and extracellular hydrophobic precursor export. These findings not only decipher the molecular basis of the epidermal barrier in blueberries but also provide high-value genetic targets for breeding cultivars with improved storability.

## Figures and Tables

**Figure 1:**
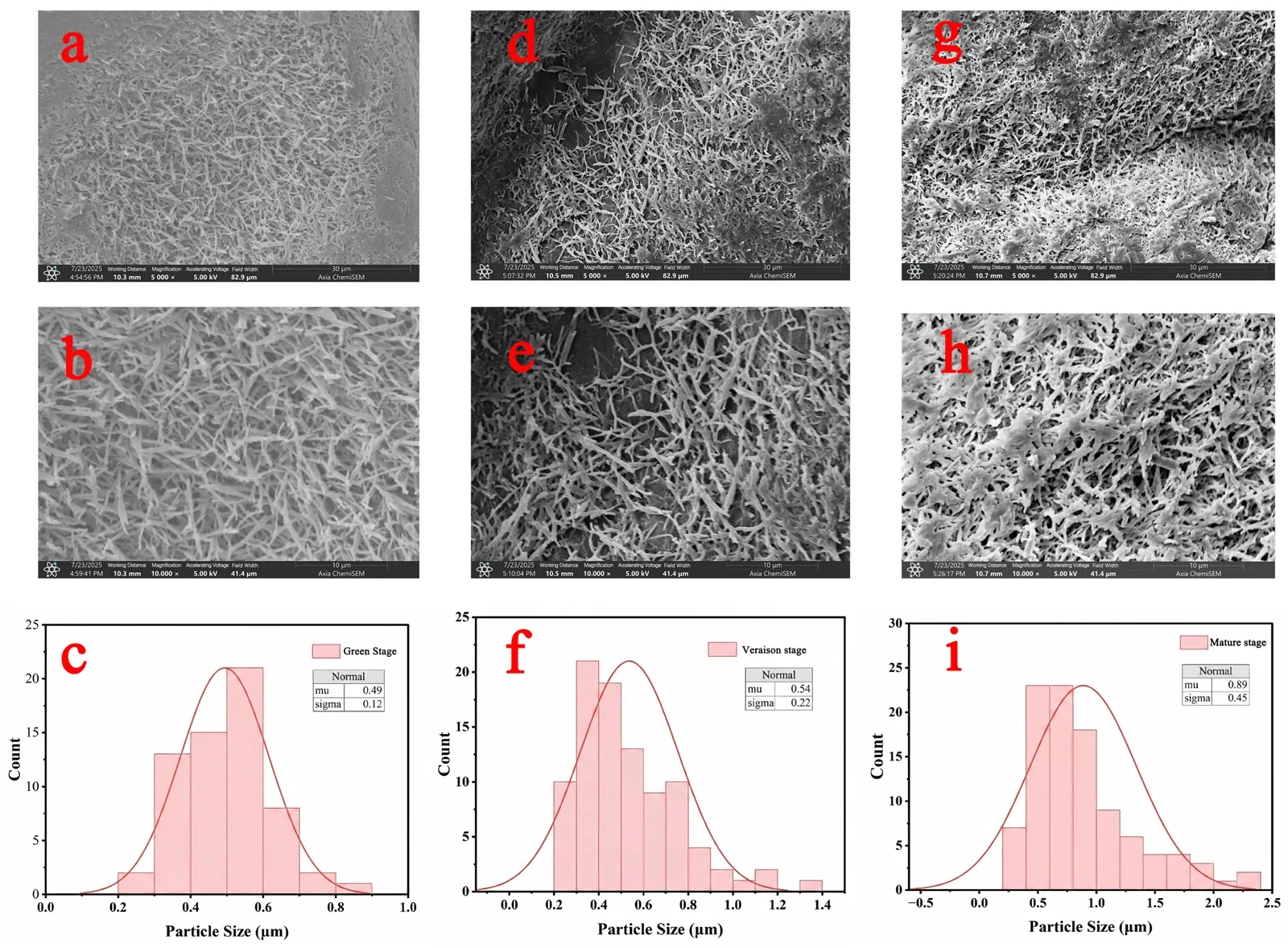
Evolution of the microstructural characteristics and crystal size distribution of epicuticular wax during blueberry fruit development. Note: (**a**,**d**,**g**) Randomly distributed fibrous wax crystals and loose network structures observed at 5000× magnification during the green, veraison, and mature stages, respectively. Scale bars = 30 μm. (**b**,**e**,**h**) Details of wax fibers and partially exposed epidermis observed under a high magnification of 10,000× during the green, veraison, and mature stages. Scale bars = 10 μm. (**c**,**f**,**i**) Frequency distribution histograms and Gaussian fitting curves of wax crystal diameters at the green, veraison, and mature stages. The red curves represent the normal distribution fits.

**Figure 2:**
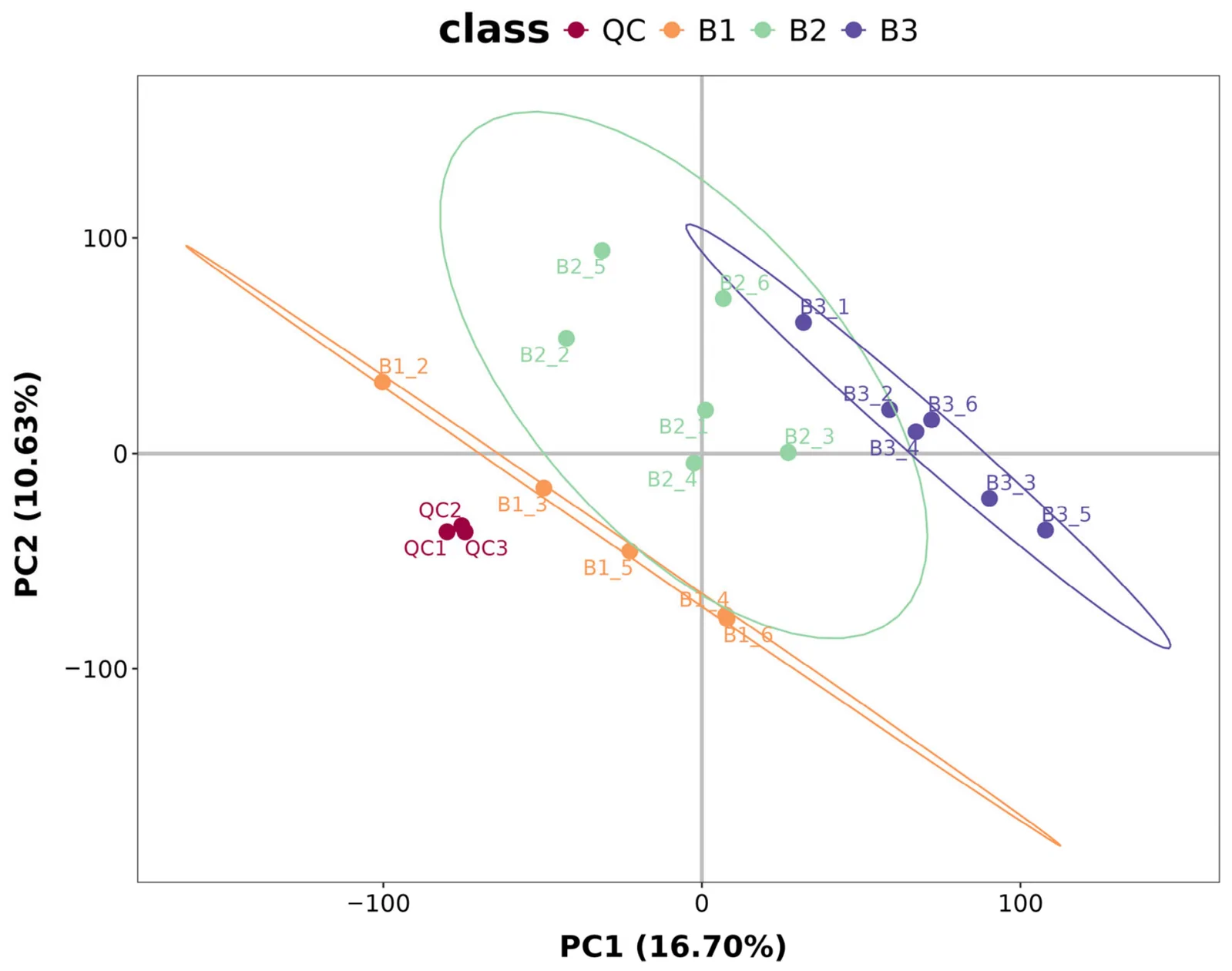
Principal component analysis (PCA) score plot of the epicuticular wax metabolome during blueberry fruit development. Note: PC1 and PC2 explained 16.70% and 10.63% of the total variance, respectively. QC, quality-control samples; B1, B2 and B3 denote the green, veraison, and mature stages. OPLS-DA model parameters are reported in Section 2.2.1.

**Figure 6:**
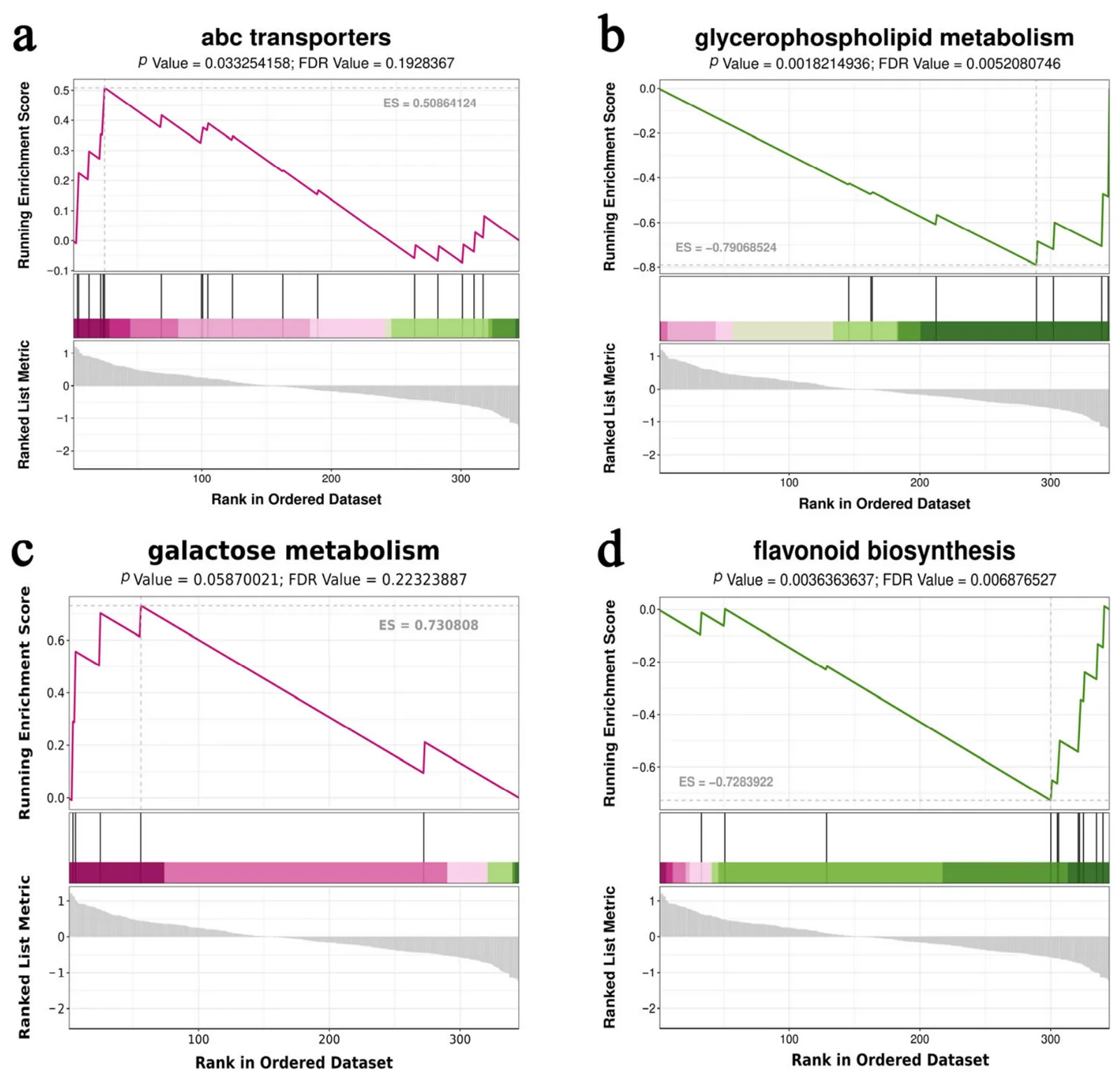
GSEA enrichment analysis of key pathways in the epicuticular wax metabolome (B3 vs. B1). Note: Panels (**a**–**d**) illustrate four key metabolic pathways significantly enriched in the epicuticular wax metabolome between the mature stage (B3) and the green stage (B1). Green curves represent the Running Enrichment Score (ES). Black vertical lines below the *x*-axis represent the positions of metabolites from each pathway in the ranked list. The color strip at the bottom of each panel ranks all detected metabolites by the ranking metric, with red indicating metabolites more abundant at the mature stage (B3) and green indicating metabolites more abundant at the green stage (B1); the vertical dashed line marks the rank position at which the running ES attains its extremum (i.e., the peak ES reported for each panel). (**a**) ABC transporters (*p* = 0.033); (**b**) glycerophospholipid metabolism (*p* = 0.0018); (**c**) galactose metabolism (*p* = 0.059); (**d**) flavonoid biosynthesis (*p* = 0.0036). Panels (**a**,**c**) showed positive enrichment, whereas panels (**b**,**d**) showed negative enrichment.

**Figure 7:**
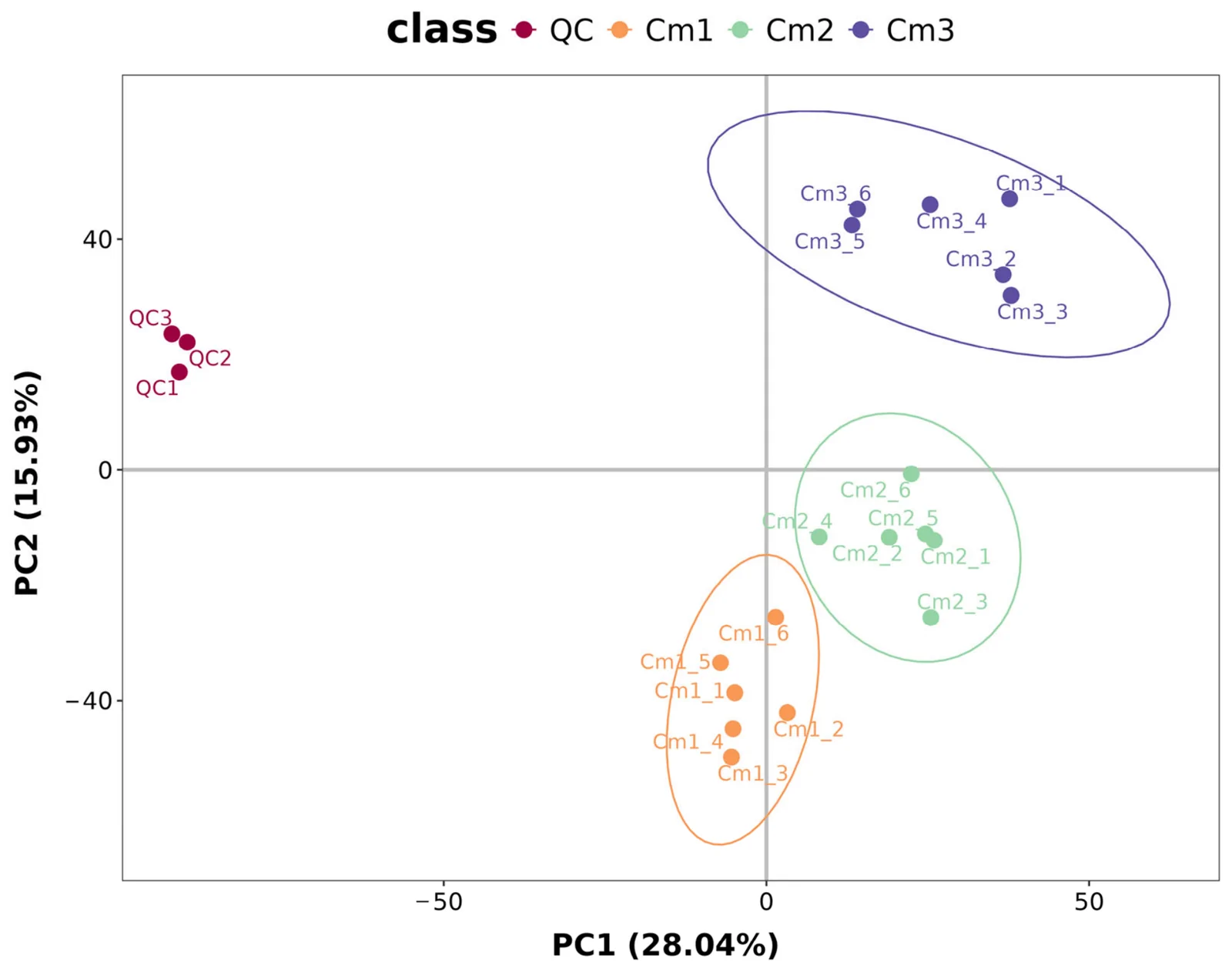
Principal component analysis (PCA) score plot of the whole-fruit metabolome during blueberry fruit development. Note: PC1 and PC2 explained 28.04% and 15.93% of the total variance, respectively. QC, quality-control samples; Cm1, Cm2 and Cm3 denote the green, veraison, and mature stages. OPLS-DA model parameters are reported in Section 2.3.1.

**Figure 8:**
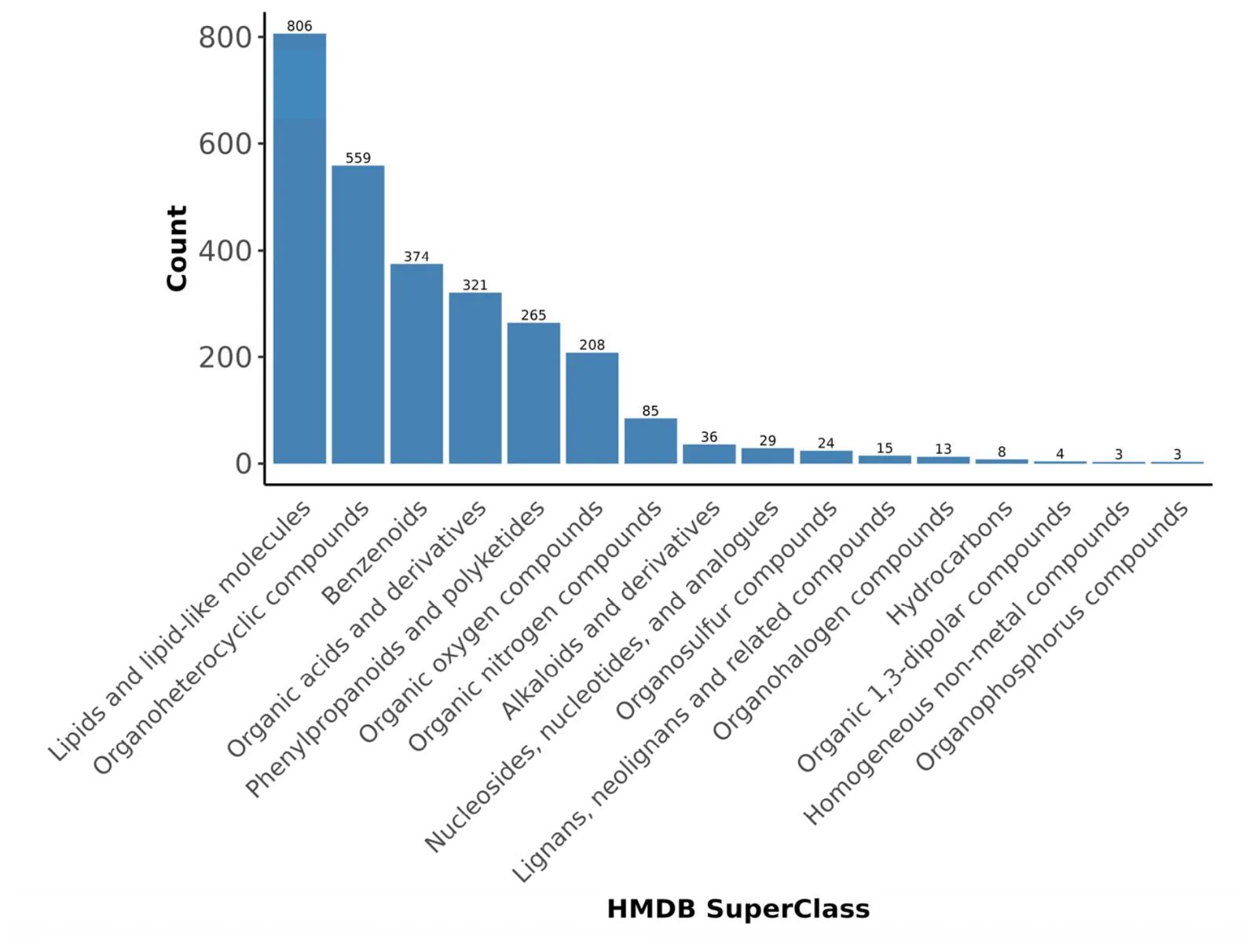
HMDB SuperClass annotation of secondary metabolites in the whole-fruit metabolome.

**Figure 9:**
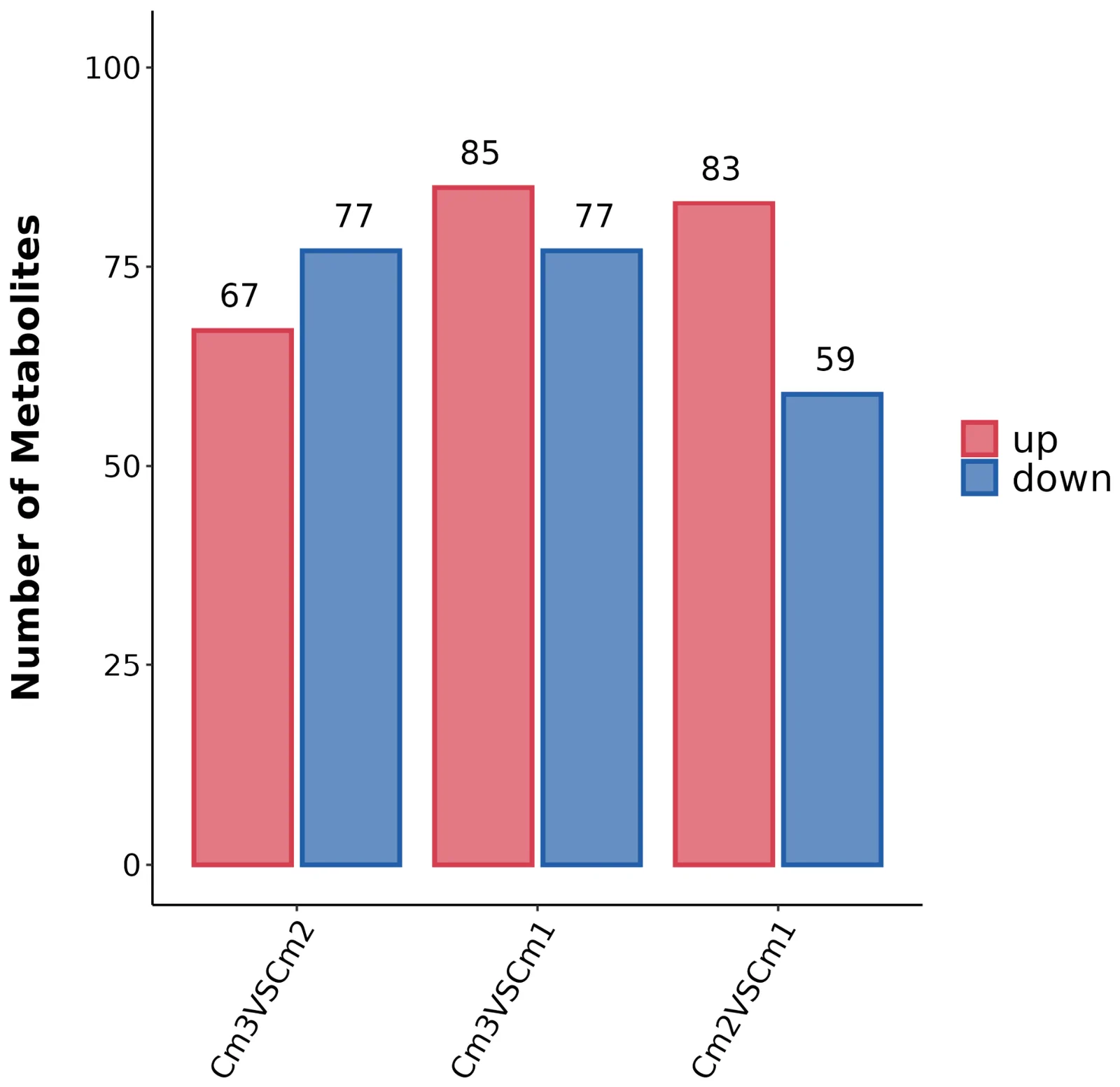
Pairwise differential metabolite analysis of the whole-fruit metabolome across developmental stages. Note: Differential metabolites were identified using variable importance in projection (VIP) > 1 from the OPLS-DA model combined with *p* < 0.05.

**Figure 10:**
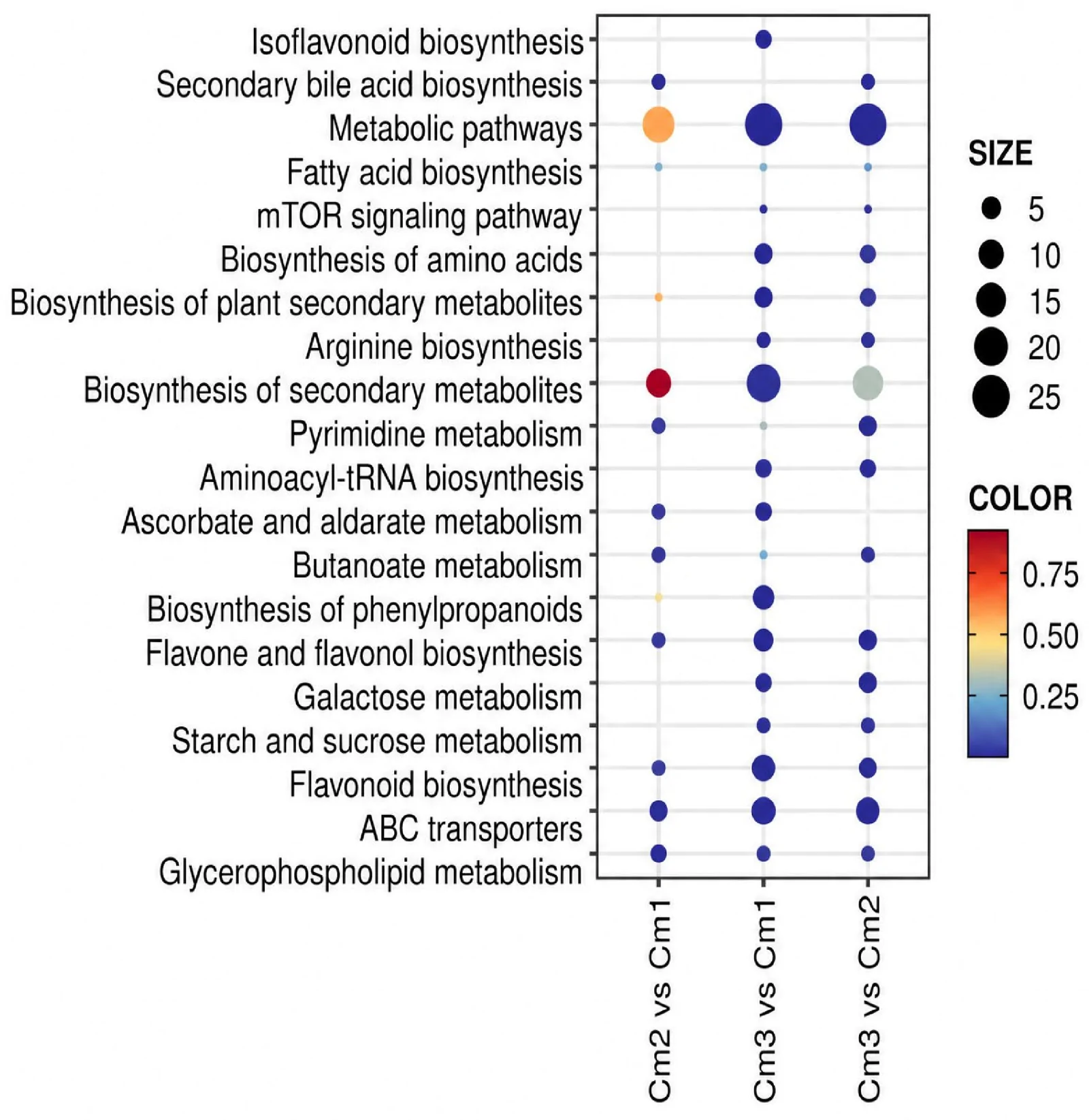
KEGG pathway enrichment analysis of the whole-fruit metabolome. Note: Bubble size and color encode the number of differential metabolites involved in each pathway and the enrichment significance, respectively.

**Figure 11:**
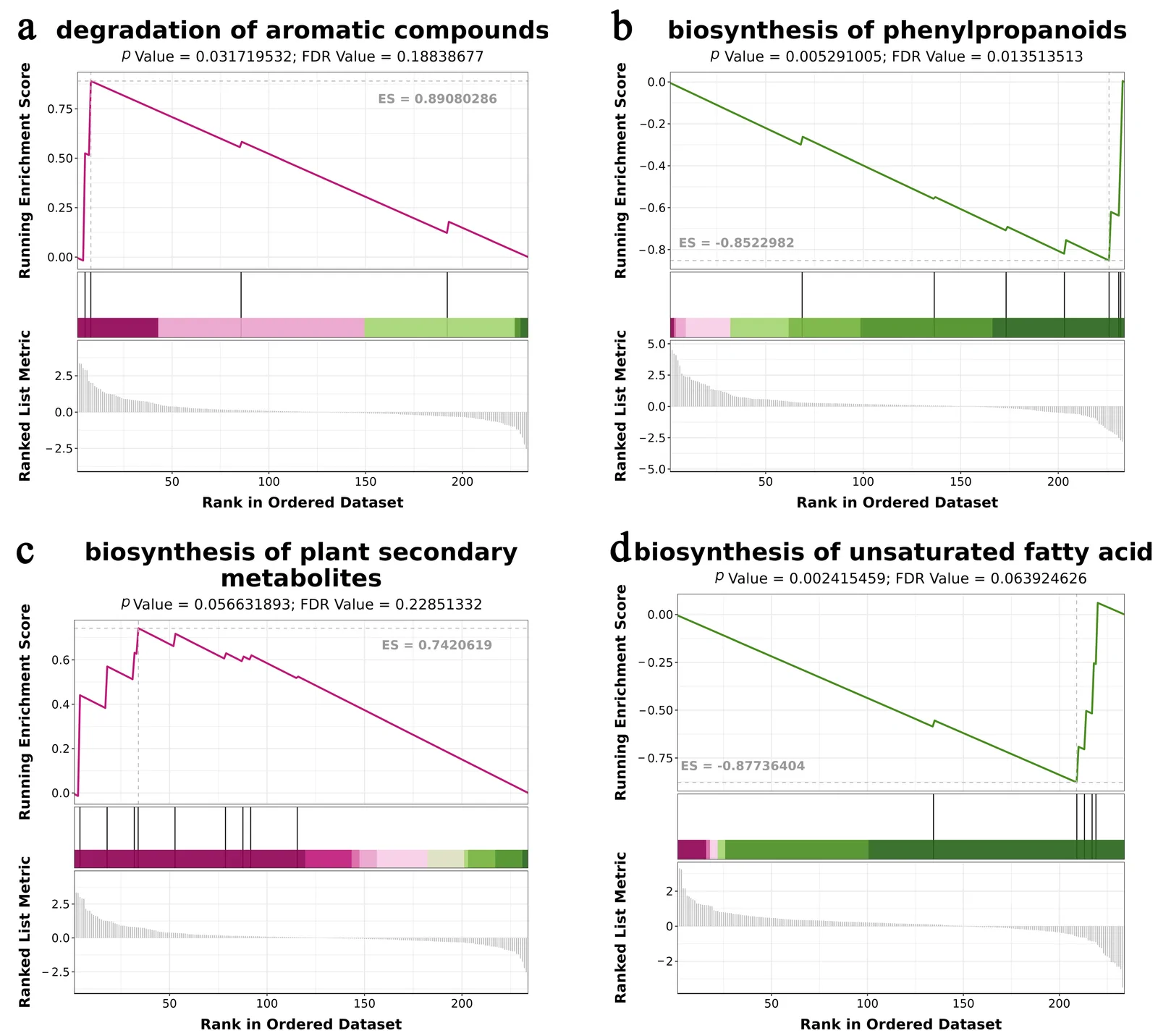
GSEA enrichment analysis of key pathways in the whole-fruit metabolome. Note: Panels (**a**–**d**) illustrate key metabolic pathways significantly enriched in blueberry fruits during the developmental transition phase (Cm2 vs. Cm1) and the mature stage (Cm3 vs. Cm1/Cm2). NES values: degradation of aromatic compounds, 1.43 (*p* < 0.05); biosynthesis of plant secondary metabolites, 1.38 (*p* = 0.057); biosynthesis of phenylpropanoids, −1.69 (*p* < 0.01); biosynthesis of unsaturated fatty acids, −1.63 (*p* < 0.01). The color strip at the bottom of each panel ranks all detected metabolites by the ranking metric of the corresponding comparison, with red indicating metabolites more abundant in the former group (Cm2 or Cm3) and green indicating metabolites more abundant in the latter group (Cm1 or Cm1/Cm2); the vertical dashed line marks the rank position at which the running ES attains its extremum (i.e., the peak ES reported for each panel).

**Figure 12:**
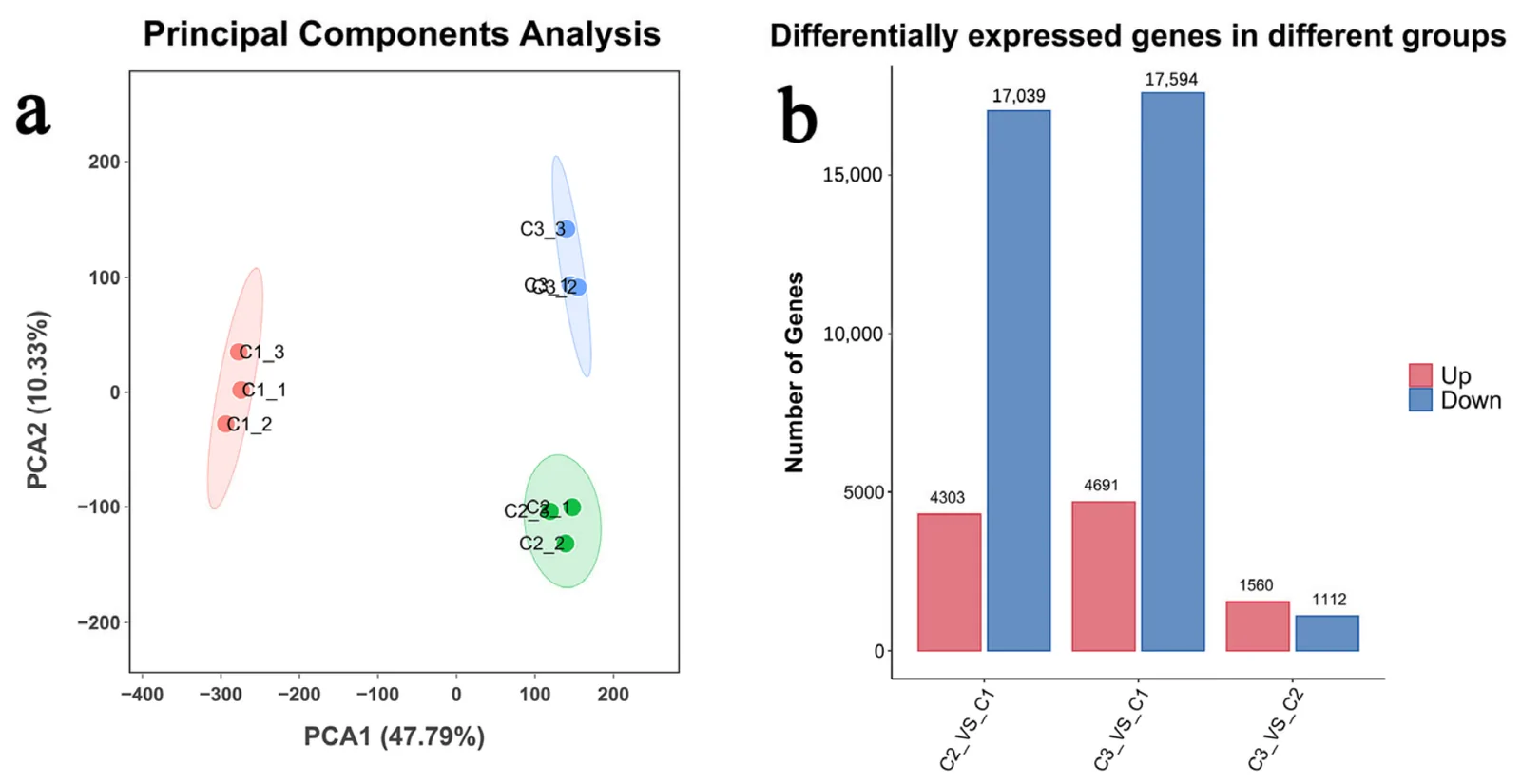
Assessment of transcriptome sequencing quality and dynamic distribution statistics of DEGs during fruit development. Note: (**a**) PCA score plot of global gene expression; PC1 and PC2 explained 47.79% and 10.33% of the total variance, respectively. (**b**) Numbers of up- and downregulated DEGs in each pairwise comparison (|log2FC| ≥ 1, *p* < 0.05). C1, C2, and C3 in the figure denote the green, veraison, and mature stages (Ct1–Ct3), respectively.

**Figure 13:**
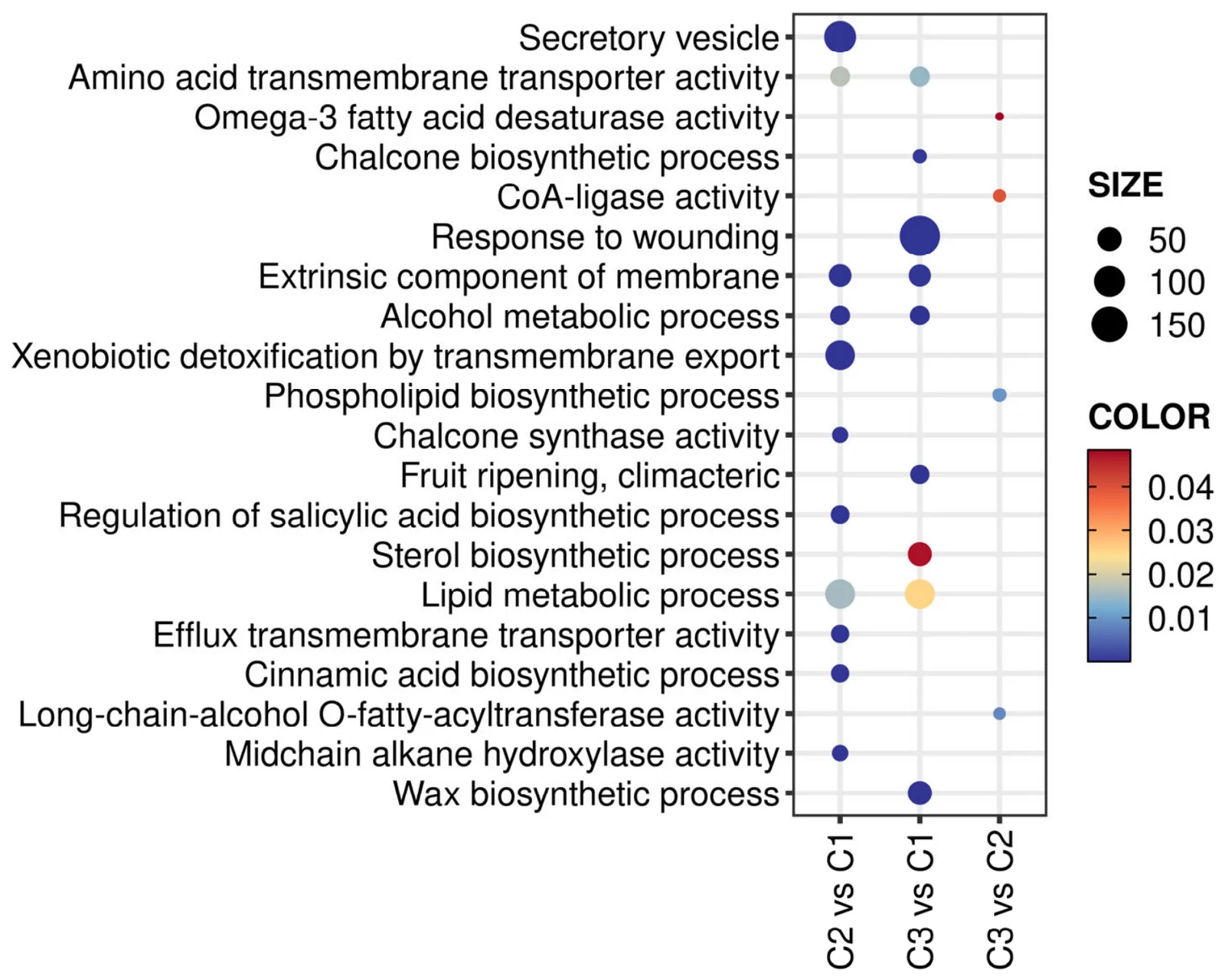
GO functional enrichment analysis of DEGs related to cuticular wax formation during fruit development. Note: GO terms with *p* < 0.05 were considered significantly enriched; circle size and color encode the number of involved DEGs and enrichment significance, respectively.

**Figure 14:**
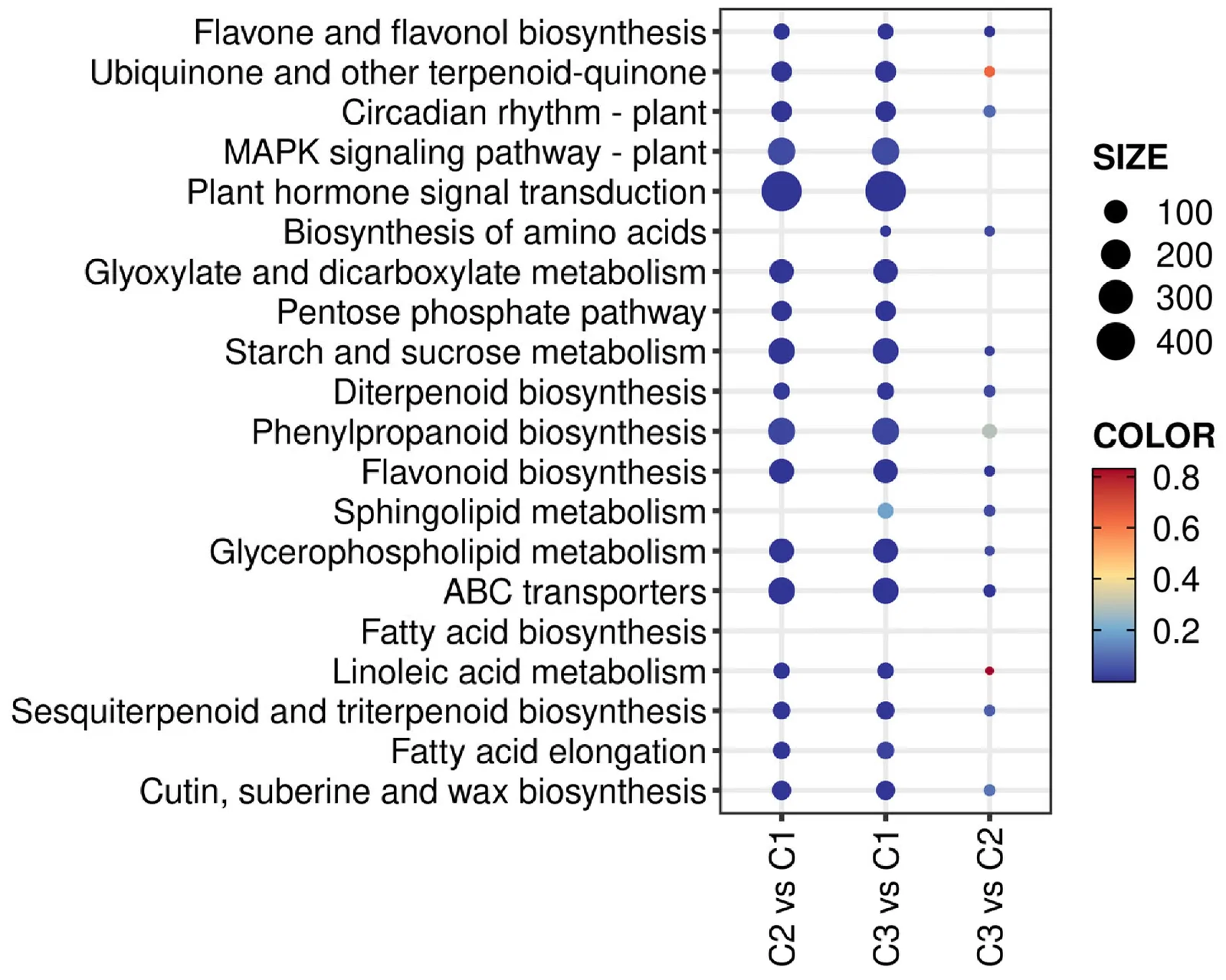
KEGG functional enrichment analysis of DEGs related to cuticular wax formation during fruit development. Note: KEGG pathways with *p* < 0.05 were considered significantly enriched; circle size and color encode the number of involved DEGs and the enrichment significance, respectively.

**Figure 15:**
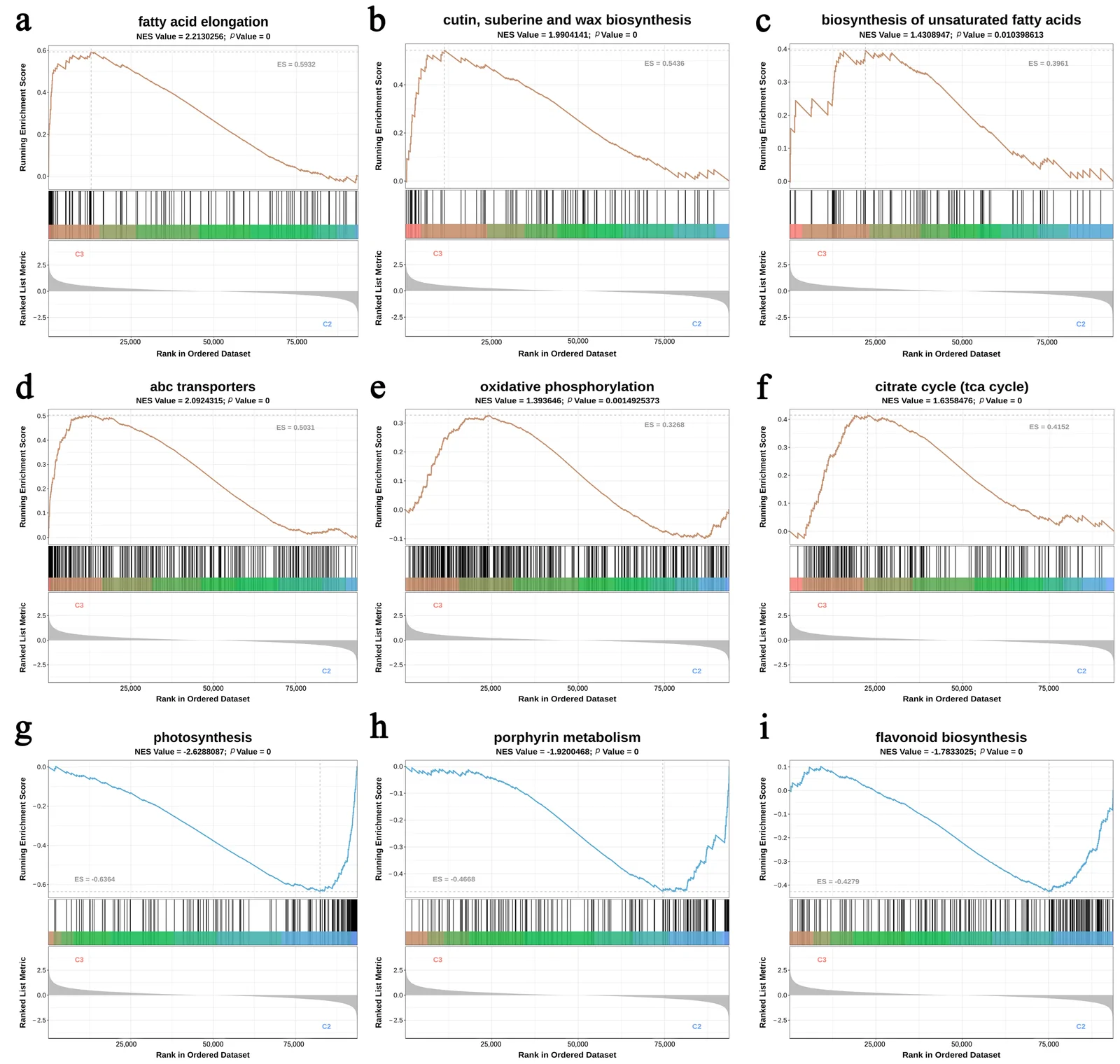
Characteristics of gene enrichment related to cuticular wax formation at the mature fruit stage (Ct3 vs. Ct2). Note: GSEA was performed against the KEGG pathway database; pathways with nominal *p* < 0.05 and FDR *q* < 0.25 were considered significantly enriched. Normalized enrichment scores (NES) and significance for each panel: (**a**) fatty acid elongation, NES = 2.21, *q* < 0.001; (**b**) cutin, suberine and wax biosynthesis, NES = 1.99, *q* < 0.001; (**c**) biosynthesis of unsaturated fatty acids, NES = 1.63, *p* = 0.003; (**d**) ABC transporters, NES = 2.09, *q* < 0.001; (**e**) oxidative phosphorylation, NES = −1.26, *p* = 0.0014; (**f**) citrate cycle (TCA cycle), NES = 1.64, *q* = 0.034; (**g**) photosynthesis, NES = −2.63, *q* < 0.001; (**h**) porphyrin metabolism, NES = −1.92, *p* < 0.001; (**i**) flavonoid biosynthesis, NES = −1.78, *q* = 0.009. Panels (**a**–**d**), (**e**,**f**) and (**g**–**i**) correspond to the lipid biosynthesis and transmembrane transport module, the energy metabolism module, and the developmental timing and functional transition module, respectively. The color strip at the bottom of each panel ranks all expressed genes by the ranking metric, with red indicating genes more highly expressed at the mature stage (Ct3) and green indicating genes more highly expressed at the veraison stage (Ct2); the vertical dashed line marks the rank position at which the running ES attains its extremum (i.e., the peak ES from which the NES is derived).

**Figure 16:**
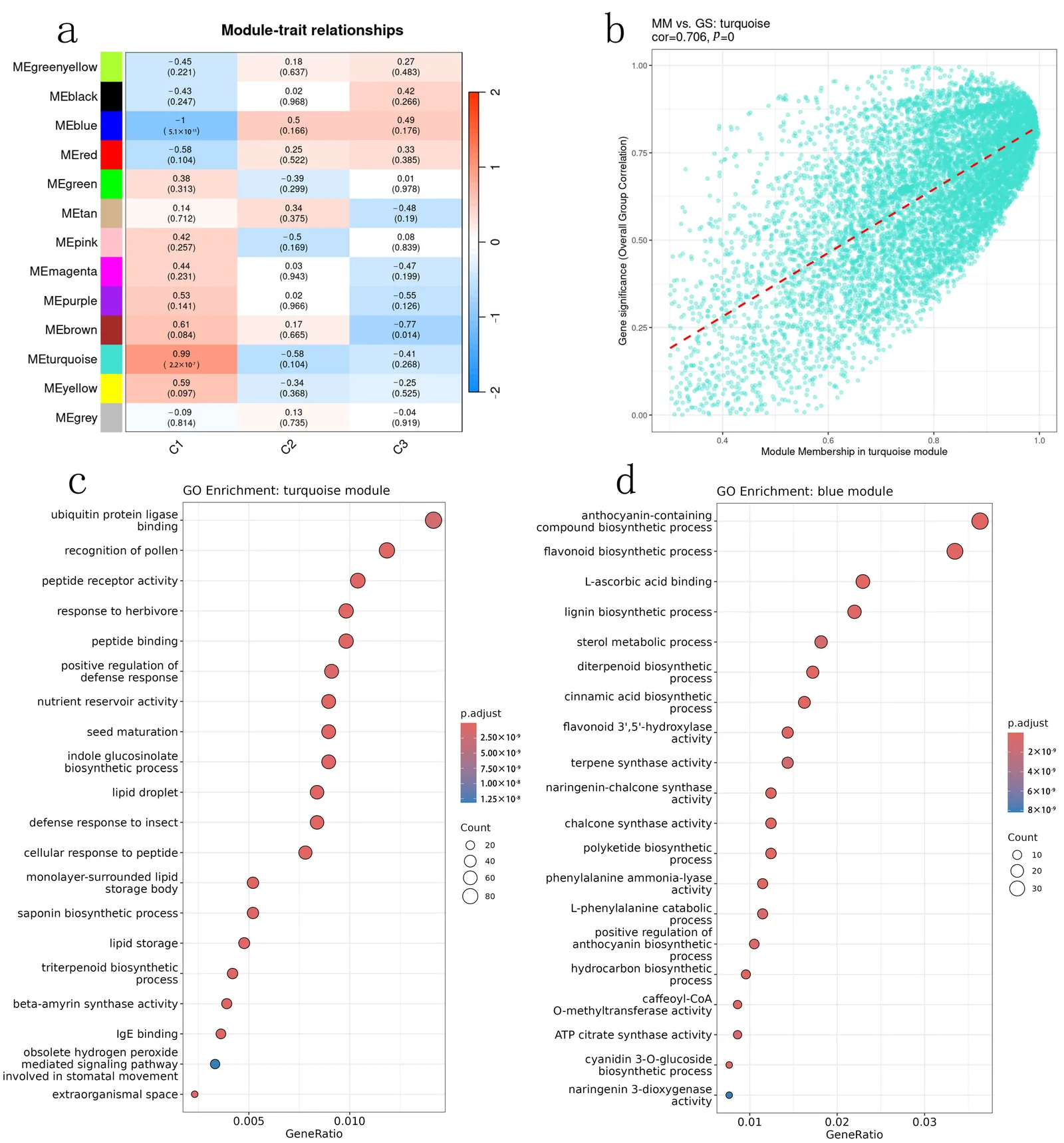
Construction of the weighted gene co-expression network (WGCNA) and identification of the topology and function of key modules during blueberry development (Ct1–Ct3). Note: (**a**) Module–trait relationship heatmap; each cell gives the correlation coefficient (upper value) and the corresponding *p*-value (in parentheses) between module eigengenes and traits. (**b**) Scatter plot of gene significance (GS) versus module membership (MM) for the Turquoise module (cor = 0.706, *p* < 1 × 10^−200^); the Blue module showed a similar and stronger GS–MM correlation (cor = 0.865). (**c**) Representative GO terms enriched in the Turquoise module. (**d**) Representative GO terms enriched in the Blue module.

**Table 1:** Experimental design and sample information for blueberry cuticular wax analysis.

Developmental Stage	Stage Description	SEM Morphology	Wax Metabolomics	Fruit Transcriptomics	Fruit Metabolomics	Measured Parameters
Green stage	Green fruit development stage	SEM-A1	B1	Ct1	Cm1	Microstructure, wax composition, gene expression
Veraison stage	Onset of fruit coloration	SEM-A2	B2	Ct2	Cm2	Microstructure, wax composition, gene expression
Mature stage	Fully mature fruit	SEM-A3	B3	Ct3	Cm3	Microstructure, wax composition, gene expression

## Data Availability

The raw transcriptome sequencing data generated in this study have been deposited in the NCBI Gene Expression Omnibus (GEO) database under the accession number GSE327820. All other data supporting the findings of this study are available within the article and its Supplementary Materials.

## Supplementary Materials

The following supporting information can be downloaded at: https://www.mdpi.com/article/10.3390/ijms27198726/s1.

## Abbreviations

The following abbreviations are used in this manuscript:

ABC	ATP-binding cassette
ABA	abscisic acid
ACP	acyl carrier protein
ATP	adenosine triphosphate
DEGs	differentially expressed genes
ESI	electrospray ionization
FDR	false discovery rate
FPKM	fragments per kilobase of transcript per million mapped reads
GC-MS	gas chromatography-mass spectrometry
GEO	Gene Expression Omnibus
GO	Gene Ontology
GS	gene significance
GSEA	gene set enrichment analysis
HMDB	Human Metabolome Database
KEGG	Kyoto Encyclopedia of Genes and Genomes
KNN	K-nearest neighbors
LASSO	least absolute shrinkage and selection operator
LC-MS	liquid chromatography-mass spectrometry
MEs	module eigengenes
MM	module membership
MS	mass spectrometry
NCBI	National Center for Biotechnology Information
NES	normalized enrichment score
OPLS-DA	orthogonal partial least squares discriminant analysis
ORA	over-representation analysis
PCA	principal component analysis
QC	quality control
qRT-PCR	quantitative real-time polymerase chain reaction
RIN	RNA integrity number
RNA-Seq	RNA sequencing
SEM	scanning electron microscopy
TOM	topological overlap matrix
UHPLC	ultra-high performance liquid chromatography
UPLC-MS/MS	ultra-high performance liquid chromatography-tandem mass spectrometry
VIP	variable importance in projection
VLCFA	very-long-chain fatty acid
WGCNA	weighted gene co-expression network analysis
